# The Effects of Erchen Decoction on Gut Microbiota and Lipid Metabolism Disorders in Zucker Diabetic Fatty Rats

**DOI:** 10.3389/fphar.2021.647529

**Published:** 2021-07-22

**Authors:** Tian Zhao, Libin Zhan, Wen Zhou, Wanxin Chen, Jintong Luo, Lijing Zhang, Zebin Weng, Chunyan Zhao, Shenlin Liu

**Affiliations:** ^1^ School of Traditional Chinese Medicine and School of Integrated Chinese and Western Medicine, Nanjing University of Chinese Medicine, Nanjing, China; ^2^ Affiliated Hospital of Nanjing University of Chinese Medicine, Nanjing, China; ^3^ Jiangsu Provincial Hospital of Traditional Chinese Medicine, Nanjing, China

**Keywords:** obesity, Erchen decoction, lipid metabolism disorders, insulin resistance, gut microbiota, short-chain fatty acids

## Abstract

Obesity is a chronic metabolic disease caused by genetic and environmental factors that has become a serious global health problem. There is evidence that gut microbiota is closely related to the occurrence and development of obesity. Erchen Decoction (ECD), a traditional Chinese medicine, has been widely used for clinical treatment and basic research of obesity and related metabolic diseases in recent years. It can significantly improve insulin resistance (IR) and lipid metabolism disorders. However, there is no microbiological study on its metabolic regulation. In this study, we investigated the effects of ECD on obesity, especially lipid metabolism and the composition and function of gut microbiota in Zucker diabetic fatty (ZDF) rats, and explored the correlation between the biomarkers of gut microbiota and metabolite and host phenotype. The results showed that ECD could reduce body weight, improve IR and lipid metabolism, and reduce the concentration of free fatty acids (FFA) released from white adipose tissue (WAT) due to excessive lipolysis by interfering with the insulin receptor substrate 1 (IRS1)/protein kinase B (AKT)/protein kinase A (PKA)/hormone-sensitive triglyceride lipase (HSL) signaling pathway in ZDF rats. Additionally, ECD gradually adjusted the overall structure of changed gut microbiota, reversed the relative abundance of six genera, and changed the function of gut microbiota by reducing the content of propionic acid, a metabolite of gut microbiota, in ZDF rats. A potentially close relationship between biomarkers, especially *Prevotella*, *Blautia*, and *Holdemania*, propionic acid and host phenotypes were demonstrated through correlation analysis. The results suggested that the beneficial effects of ECD on obesity, especially lipid metabolism disorders, are related to the regulation of gut microbiota in ZDF rats. This provides a basis for further research on the mechanism and clinical application of ECD to improve obesity via gut microbiota.

**GRAPHICAL ABSTRACT:**
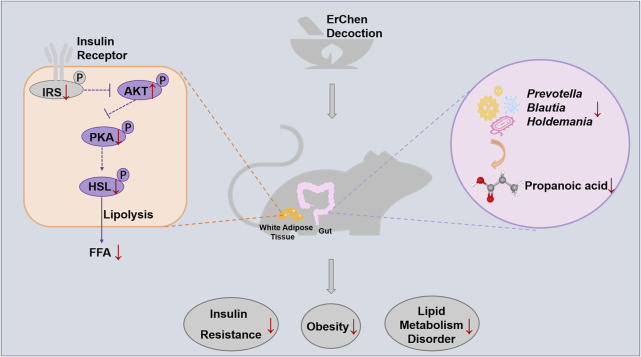
Erchen Decoction could inhibit excessive lipolysis and improve lipid metabolism disorders by regulating the IRS1/AKT/PKA/HSL signaling pathway in white adipose tissue of ZDF rats. The delay in developing obesity was related to changes in gut microbiota composition and function in ZDF rats.

## Introduction

With the improvement of living standards and changes in lifestyles, the number of obese people is increasing sharply. The latest data has showed that, there were more than 1.9 billion adults worldwide who were overweight (about 39% of the total), and 650 million who were obese (about 13% of the total). 38 million children under the age of five were overweight or obese^1^. Obesity is an important risk factor for many metabolic diseases (Saltiel and Olefsky, 2017), cardiovascular diseases (Luo et al., 2018), and even certain types of cancers (Majchrzak et al., 2019). At present, obesity, especially primary obesity, is considered to be a common disease. How to effectively prevent obesity and reduce the occurrence and development of related diseases has become a major research focus.

Adipose tissue is a main depot for storing and releasing energy and plays a key role in energy homeostasis, especially lipid metabolism balance. Dysfunction and metabolic disorder in adipose tissue is a characteristic pathological change in obesity and an important cause of local inflammation and systemic insulin resistance (IR) (Caprio et al., 2017). Insulin signaling is of crucial importance for maintaining adipose tissue function (Czech, 2017), whether from circulation or central insulin signaling (Scherer et al., 2011). Adipose tissue IR, especially an impaired insulin-signaling pathway, affects the key enzymes of lipolysis (Frühbeck et al., 2014), which leads to enhanced lipolysis as an important manifestation of metabolic disorders in adipose tissue.

Erchen Decoction (ECD), a traditional Chinese medicine formula, was first recorded in the Taiping Huimin Formula Bureau in the Song Dynasty, and is mainly used to treat phlegm dampness syndrome due to spleen dysfunction and dampness accumulation. Modern studies have found that ECD has beneficial weight loss, anti-inflammatory, and anti-oxidation effects, and significantly improves decreased insulin sensitivity (Zhang et al., 2017) and glucose and lipid metabolism disorders, especially lipid metabolism in metabolic diseases (Gao et al., 2015; Ding et al., 2018; Zhang et al., 2020b; Lee et al., 2020). In recent years, growing evidence has linked changes in gut microbiota with insulin sensitivity (Pedersen et al., 2016) and lipid metabolism (Kindt et al., 2018), and is now a target for obesity treatment (Maruvada et al., 2017). The potential therapeutic mechanism of Chinese herbal medicines to ameliorate related metabolic diseases by improving the gut microbiota is also gradually being discovered (Gong et al., 2020). Previous studies have reported that the metabolism improvement of main traditional Chinese medicines (such as *Wolfiporia extensa* (Peck) Ginns (syn. *Poria cocos* (Schwein.) F.A.Wolf) and *Zingiber Officinale* Roscoe (Wang et al., 2020)) and their extracts (such as *Citrus reticulata* Blanco extract (Zhang et al., 2020c)) and active ingredients (such as glycyrrhiza polysaccharide (Zhang et al., 2018)) in ECD was closely related to the modulation of gut microbiota. A series of studies have been conducted on the effects by which ECD improves obesity. However, the role of ECD as a compound recipe in gut microbiota and whether the effect of ECD on improving IR or lipid metabolism disorders is related to changes in intestinal microbiota are still unclear.

The aim of this study was to observe whether ECD intervention could induce changes in IR and lipid metabolism disorders, delay the development of obesity, and affect the composition and function of gut microbiota in Zucker diabetic fatty (ZDF) rats, a spontaneous obesity model. More importantly, our goal was to determine the underlying correlation between the biological effects of ECD and the changes of gut microbiota and to provide a theoretical basis by which ECD improves obesity and related metabolic diseases via a gut microbiological mechanism.

## Materials and Methods

### Preparation of ECD

ECD is composed of six components, as shown in Table 1. All herbs were purchased from Sanyue Chinese Traditional Medicine Co., Ltd. (Nantong, China) and prepared according to the Chinese Pharmacopeia method (Chinese Pharmacopoeia Commission, 2015). The medicines were soaked in eight weight/volume (1:8, w/v) distilled water for 2 h. After boiling on high heat, they were simmered at low heat for 30 min. They were extracted twice, and the filtrate combined and concentrated until the final crude drug concentration was 0.23 g/ml for low dose, 0.46 g/ml for medium dose, and 0.92 g/ml for high dose. The medium dose is clinically effective dose of ECD. The samples were stored in a refrigerator at 4°C.

**TABLE 1:** The components of ECD.

Herbal name	Botanical Latin name	Place of origin	Part used	Amount used
Ban-Xia	*Pinellia ternata* (Thunb.) Makino	Jiangsu	dried tuber	15 g
Chen-Pi	*Citrus × aurantium* L.	Zhejiang	dried mature pericarp	15 g
Fu-Ling	*Wolfiporia extensa* (Peck) *Ginns* (syn. *Poria cocos* (Schwein.) F.A.Wolf)		dried sclerotia	9 g
Gan-Cao	*Glycyrrhiza uralensis* Fisch. ex DC.	Gansu	dried root and rhizome	4.5 g
Sheng-Jiang	*Zingiber Officinale* Roscoe	Jiangsu	fresh rhizome	7 pieces
Wu-Mei	*Prunus mume* (Siebold) Siebold and Zucc.	Fujian	dry near-mature fruit	1 piece

### Chemical Composition of ECD Samples

High performance liquid chromatography (HPLC) was performed on a Waters 2,695 system (Waters Corporation, Milford, MA, United States), consisting of a binary solvent delivery manager, an auto-sampler, and a PDA detector. Chromatographic separations were performed on an Alltima C18 column (250 × 4.6 mm, 5 μm). Flow rate and column temperature were set at 1 ml min^−1^ and 30°C, respectively. A mobile phase system consisting of 0.1% formic acid in H_2_O (A)-acetonitrile (B) was applied with the following gradient program: 0–5 min, 95% A; 5–15 min, 95–75% A; 15–24 min, 75% A; 24–29 min, 75–65% A; 29–34 min, 65% A; 34–39 min, 65–55% A; 39–44 min, 55–50% A; 44–50 min, 50% A; 50–55 min, 50–30% A; 55–60 min, 30% A; 60–70 min, 30–10% A; 70–75 min, 10% A; 75–80 min, 10–0% A; 80–83 min, 0% A; 83–86 min, 0–95% A; 86–90 min, 95% A. The injection volume was 10 μL. Ultraperformance liquid chromatography-electrospray ionization-quadrupole-time of flight-mass spectrometry (UHPLC-ESI-Q-TOF-MS) was also performed on ECD samples. Details of the detection method are described in the supplementary materials.

### Animal Model

We used 32 ZDF rats (*Fa/Fa*) with body weights of 130 ± 10 g, and six Zucker lean (ZL) rats (*Fa/+*) with body weights of 102 ± 12 g. All rats were 5 weeks old, male, with animal quality certificate No. SCXK (Beijing) 2016–0,006 provided by Vital River Laboratories (Beijing, China). They were raised in the specific pathogen-free animal experiment center at Nanjing University of Chinese medicine (Nanjing, China) at a temperature of 24 ± 2°C, humidity of 65 ± 5%, light/dark cycle of 12 h/12 h, and were provided food and water *ad libitum*. All animal experiments were approved by the Animal Ethics Committee of Nanjing University of Chinese Medicine (approval No. 201909A017). All studies were conducted in accordance with the recommendations of Guide for the Care and Use of Laboratory Animals.

### Experimental Design

After adaptive feeding, rats were randomly divided into five groups: control group (L, *n* = 6), model group (Z, *n* = 8), ECD low-dose group (EC-L, *n* = 8), medium-dose group (EC-M, *n* = 8), and high-dose group (EC-H, *n* = 8). Group L was fed with a normal diet (MD17121, Mediscience, China), and the others were given formula feed (Purina#5008, Lab diet, United States). Dietary composition is shown in Supplementary Table 1. From 5 to 9 weeks old, ECD treatment groups (EC-L, EC-M, and EC-H groups) were orally administered the low (2.28 g/kg), medium (4.57 g/kg), or high (9.14 g/kg) doses of ECD, and the L and Z groups were given high-pressure-sterilized water instead of ECD once a day with a volume of 1 ml/100 g. These dosages were calculated from the equivalent conversion of the body surface area between animals and humans.

Fresh fecal samples were collected into sterile tubes, avoiding contact with skin or urine of rats, at the end of adaptive feeding (5-week-old rats) and before the end of the experiment (9-week-old rats), then stored at −80°C before processing for 16S rRNA gene sequencing. The body weights, abdominal circumferences, and food intakes of rats in all five groups were measured weekly. At the age of 9 weeks, an insulin tolerance test (ITT) was performed by intraperitoneal injection of insulin (5 U/kg) after fasting for 6 h, and the area under the curve (AUC) was calculated.

After the experiment, the rats were fasted for 12 h and then anesthetized with isoflurane. Blood was taken from the abdominal aorta. The supernatant was collected after centrifugation at 4°C and 180 g for 10 min, and the levels of total cholesterol (TC), high-density lipoprotein cholesterol (HDL-C), low-density lipoprotein cholesterol (LDL-C), and triglycerides (TG) also with alanine aminotransferase (ALT), aspartate aminotransferase (AST), blood urea nitrogen (BUN) and creatinine (Cr) were measured by an automatic biochemical analyzer (Chemray 240, Rayto, China). Fasting serum insulin levels were determined by enzyme-linked immunosorbent assay (10–1,250–01, Mercodia, Sweden), and the Homeostasis Model Assessment-Insulin Resistance (HOMA-IR) index was calculated as follows: HOMA-IR = fasting plasma glucose (mmol/L) × fasting serum insulin (mIU/L)/22.5 (Matthews et al., 1985). The remaining samples were used for determination of fasting serum free fatty acids (FFA). The weights of perirenal WAT and epididymal WAT were measured, and the fat body ratio was calculated as follows: Fat body ratio = (perirenal or epididymal) WAT weight (mg)/body weight (g) × 100%; Total fat body ratio = (perirenal + epididymal) WAT weight (mg)/body weight (g) × 100%. The epididymal WAT from the same part of each rat was collected for hematoxylin-eosin (HE) staining and western blotting. Cecal contents (fresh feces in the cecum) were collected for targeted metabolomics analysis. EC-M group was used as the representative of EC groups for subsequent HE staining, western blotting, FFA determination, gut microbiota sequencing, and short-chain fatty acids (SCFAs) content detection. Except the paraformaldehyde fixed WAT was stored at 4°C, all samples were stored at −80°C.

### HE Staining

To detect the difference of cell morphology in WAT, the three most representative rats in groups L, Z, and EC were respectively selected and their WAT were dehydrated and embedded, and then prepared into 5-µM paraffin sections (RM2245, Leica, Germany). After stained with HE staining solution (R20570-2, Yuanye, China), WAT was observed and photographed using a microscope (BX53, Olympus, Japan).

### Western Blotting and FFA Determination

The four most representative rats in groups L, Z, and EC were respectively selected for western blotting and FFA determination. Epididymal WAT samples in three groups were homogenized in RIPA buffer (P0012B, Beyotime, Beijing, China) supplemented with a mixture of 100 × protease inhibitor cocktail (5871s, CST, United States) and 100 × phosphatase inhibitor cocktail (5870s, CST, United States) to obtain their protein samples. The same amounts of protein samples were subjected to sodium dodecyl sulfate-polyacrylamide gel electrophoresis (SDS-PAGE) and blotted with the following antibodies: phospho-Insulin Receptor Substrate 1 (IRS1) (Ser307) (#2381, CST, United States, 1:1,000), IRS1 (ab52167, Abcam, United Kingdom, 1:500), phospho-Protein Kinase B (AKT) (Ser473) (4058S, CST, United States, 1:1,000), AKT (9272S, CST, United States, 1:1,000), phospho-Protein Kinase A (PKA) α/β/γ (Thr197) (ab75991, Abcam, United Kingdom, 1:5,000), PKA α/β/γ (SC-390548, Santa Cruz, United States, 1:1,000), Phospho-hormone-sensitive triglyceride lipase (HSL) (Ser563) (AF2350, Affinity, United States, 1:2000), HSL (AF6403, Affinity, United States, 1:2000), adipose triglyceride lipase (ATGL) (A6245, ABclonal, 1:1,000) and *β*-actin (3700S, CST, United States, 1:1,000). The membranes were incubated with secondary antibodies conjugated to HRP (BA-1054/BA1050, Boster, Hubei, China, 1:2000). The immunoreactive bands were treated with chemiluminescence solution (ECL, Tanon, Shanghai, China) and detected by X-ray films. The blots were visualized with an Amersham Imager 600 (General Electric Company, United States).

According to the instruction of the determination kit (A042-2-1, Jiancheng, China), the concentrations of FFA in the serum samples of rats in groups L, Z, and EC were detected.

### Gut Microbiota Sequencing and Data Analysis

The fecal samples of rats in L, Z, and EC groups at 5 and 9 weeks old were sequenced for the 16S rRNA gene (Shanghai Personal Biotechnology Co., Ltd., Shanghai, China). According to the manufacturer’s protocol, total microbial DNA was extracted from stool samples, and DNA was quantified by a Nanodrop. The quality of DNA extraction was detected by 1.2% agarose gel electrophoresis. The V3-V4 region of the 16S rRNA gene was amplified by polymerase Chain Reaction (PCR). The amplified products were quantified by fluorescence (Microplate reader, BioTek, FLx800), and the samples were mixed according to the corresponding proportions. The sequencing Library (TruSeq Nano DNA LT Library Prep Kit, Illumina company) was prepared, and double-ended sequencing (MiSeq PE300 sequencer) was performed with a Miseq Regent Kit V3 (600 cycles).

The analysis was carried out using Quantitative Insights into Microbial Ecology (QIIME2) and R language ggplot2 package software. The sequence denoising was performed by a DADA2 analysis process (Callahan et al., 2016). According to the distribution of amplitude sequence variables (ASVs) among the groups, the Simpson index at 5 and 9 weeks of age was evaluated to characterize alpha diversity, and a box plot was drawn using R script. The differences in beta diversity at 5 and 9 weeks of age were evaluated by principal coordinates analysis (PCoA) based on unweighted UniFrac distance, a classical multidimensional scaling (cMDScale) analysis method (Ramette, 2007). Sample two-dimensional sorting graphs of PCoA were drawn by R script, and the significance of the differences was evaluated by adonis analysis. The number of common and unique ASVs between groups was shown by a Venn diagram. At the level of taxonomic composition, species at 5 and 9 weeks of age in each group was displayed at the phylum and genus levels to understand the overall microbial composition. At the genus level, the UPGMA algorithm was carried out to perform hierarchical clustering analysis based on the Bray-Curtis distance matrix to show the similarity of the microbial composition among groups. Linear discriminant analysis (LDA) effect size (LEfSe) analysis, a nonparametric Kruskal-Wallis and Wilcoxon rank sum test combined with LDA effect size (Segata et al., 2011), was applied to explore the difference between groups at 5 and 9 weeks of age, and measure the changes in microbiota during the development of obesity and ECD treatment. An LDA value distribution histogram was used to show the species significantly enriched and their degree of importance. A cladogram was constructed to display the taxonomic hierarchical distribution of biomarkers in each group. Random forest analysis was applied to show the order of importance of biomarkers among groups at 9 weeks of age. The functional potential was predicted and analyzed based on Phylogenetic Investigation of Communities by Reconstruction of Unobserved States (PICRUSt) 2. The abundance of secondary functional pathways in the KEGG pathway database (http://www.genome.jp/kegg/pathway.html) was calculated for gut microbiota of 9-week-old rats. The functional units were identified by PCoA based on Bray-Curtis similarity, and differential metabolic pathways were predicted by metagenomeSeq. Spearman correlation analysis was used to determine the correlation between biomarkers and differential metabolic pathways. A heat map was constructed to investigate the potential relationship between the biomarkers and host phenotype.

The raw sequences of Miseq sequences from 44 fecal samples of rats have been submitted to NCBI Project under accession number PRJNA686642 with NCBI Sequence Read Archive under accession number SRP298569.

### SCFAs Analysis

The targeted metabolism technology, ultraperformance liquid chromatography-tandem mass spectrometry (UPLC-MS/MS), was used to quantitatively detect SCFAs in the cecal contents of L, Z, and EC groups (Metabo-Profile, Shanghai, China). According to the manufacturer’s protocol, approximately 10 mg of sample was put in a 1.5 ml tube, and 25 μL of water and 185 μL of acetonitrile:methanol (8:2) was added to extract metabolites. After high-speed centrifugation (18,000 *g*, 20 min), 15 μL of internal standard was added to the 135 μL supernatant, which was aliquoted and diluted. UPLC-MS/MS (Waters ACQUITY UPLC-Xevo TQ-S, Waters Corp., Milford, MA, United States) was used for SCFA detection. TargetLynx software (Waters Corp., Milford, MA, United States) was used to process the original data files generated by UPLC-MS/MS, and the peaks of each metabolite were integrated, calibrated, and quantified. Partial least squares discrimination analysis (PLS-DA) was applied to show the composition of SCFAs among groups. Integrated Metabolomic Analysis Platform v1.0 (Metabo-Profile, Shanghai, China) was used for statistical analysis. A heat map was constructed to show the potential relationship between the biomarker and host phenotype.

### Statistical Analysis

The data of ZDF rat phenotypes was expressed as means ± standard error of the mean (SEM). The statistical differences between groups were evaluated by analysis of variance (ANOVA) using GraphPad Prism 8.0 software (GraphPad, La Jolla, CA, United States), and the specific analysis method is shown in the legend of each figure. ImageJ v1.8.0 (Rawak Software Inc., Stuttgart, Germany) was used to analyze the number and cross-sectional area of adipocytes in WAT. The target protein bands were quantified with ImageQuant TL 1D software (GE Healthcare, United States). Spearman correlation analysis was conducted to evaluate correlations between the biomarkers in gut microbiota and SCFAs and host phenotype. Significant differences were accepted at *p* values of <0.05.

## Results

### The Chemical Composition of ECD

**FIGURE 1:**
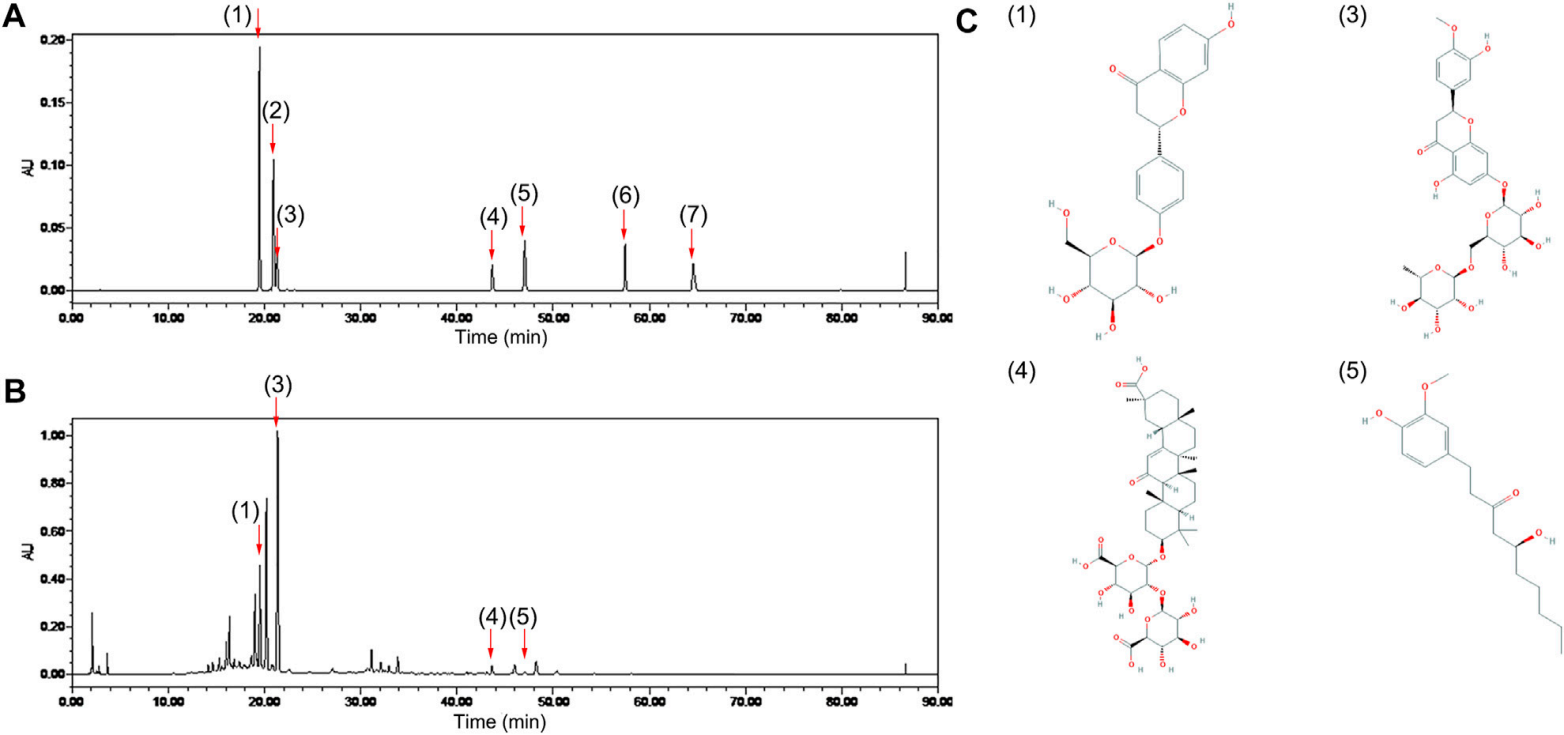
Chemical composition of ECD. Chromatogram of **(A)** mixed standards and **(B)** ECD sample by HPLC. **(C)** Chemical structures of four components in ECD sample. 1) liquiritin, 2) naringin, 3) hesperidin, 4) glycyrrhizic acid, 5) 6-gingerol, 6) 8-gingerol, 7) 10-gingerol in each figure.

A characteristic HPLC chromatogram of an ECD sample is shown in Figure 1. ECD contained four compounds, liquiritin, hesperidin, glycyrrhizic acid, and 6-gingerol, which are flavonoids, saponins, and phenols, and is basically consistent with the results of previous studies (Lee et al., 2020). UHPLC-ESI-Q-TOF-MS total ion chromatogram and results of ECD sample are shown in Supplementary Figure 1 and Supplementary Table 2. One hundred and twenty-six compounds in the positive ion mode and 20 compounds in the negative ion mode were detected, including naringin and 8-gingerol that were not detected by HPLC. Nobiletin was found in the positive ion mode, and five compounds were found in both the positive ion and negative ion mode. Previous studies have predicted that hesperidin, naringin, nobiletin, glycyrrhizic acid, and 6-gingerol might be the main bioactive components and medicinal material bases of ECD intervention in metabolic diseases (Lee et al., 2018).

### ECD Delayed the Development of Obesity in ZDF Rats

**FIGURE 2:**
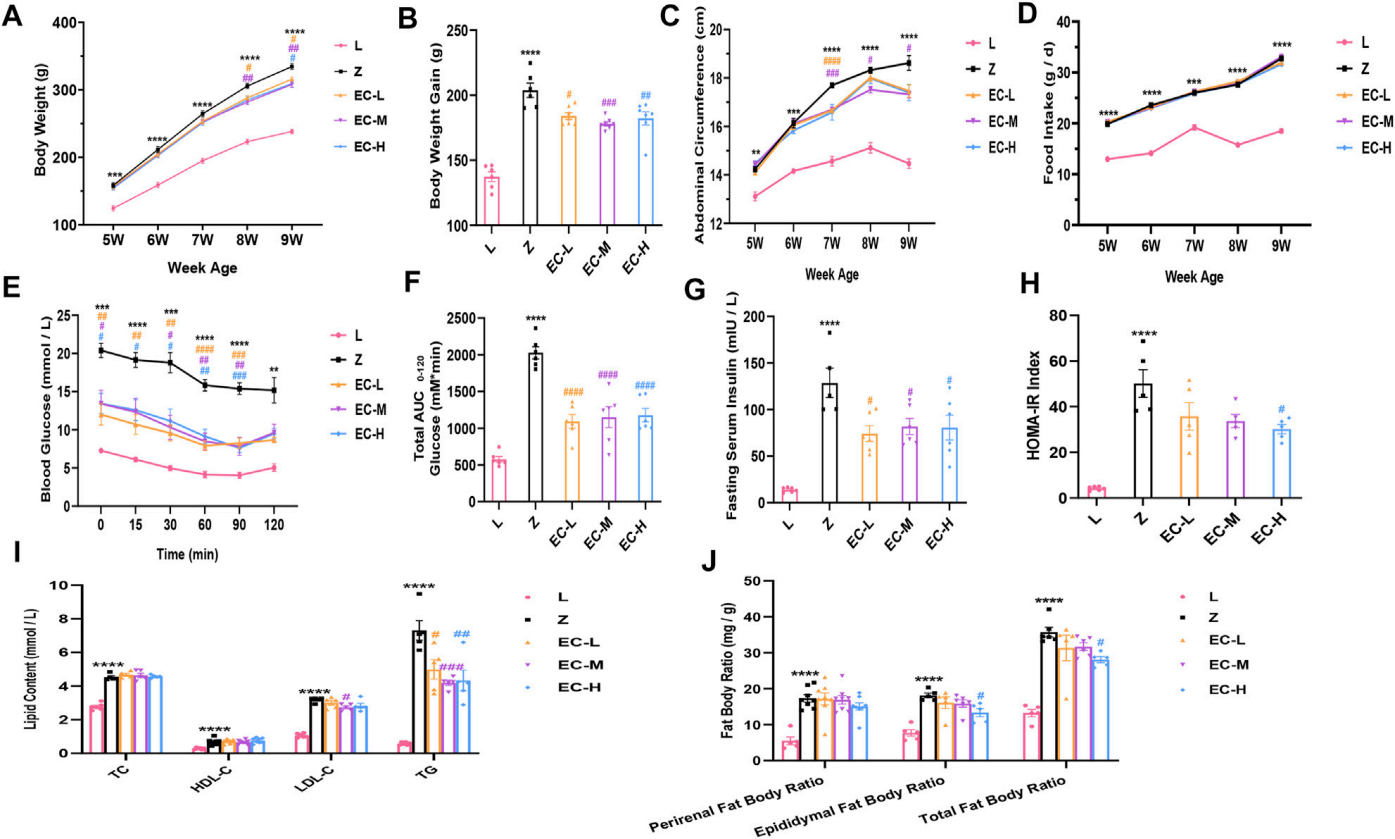
ECD delayed the development of obesity in ZDF rats. **(A)** Weekly weight change. **(B)** Weight gain at 9 weeks of age. **(C)** Weekly abdominal circumference change. **(D)** Weekly food intake change. **(E)** Blood glucose levels during ITT. **(F)** AUC based on ITT data. **(G)** Fasting serum insulin levels. **(H)** HOMA-IR index. **(I)** Perirenal, epididymal, and total fat body ratios. **(J)** Serum TC, HDL-C, LDL-C, and TG levels at 9 weeks of age. Data are expressed as means ± SEM (*n* = 5–8. Z vs. L, ^**^ *p* < 0.01, ^***^ *p* < 0.001, ^****^ *p* < 0.0001; EC-L, EC-M, and EC-H vs. Z, ^#^ *p* < 0.05, ^##^ *p* < 0.01, ^###^ *p* < 0.001, ^####^ *p* < 0.0001). (A), (C), (D), and (E) were analyzed by two-way ANOVA, the rest were analyzed by one-way ANOVA.

To observe the effects of ECD on obesity in ZDF rats, we compared the changes of body weight, abdominal circumference, and food intake in the five groups. The results revealed that the difference of body weight age-dependently increased in group Z comparison to group L. ECD treatment notably reduced the body weight of rats at 8 weeks old. At 9 weeks of age, the body weight gain compared with the baseline of the ECD-treated groups was significantly lower than that of group Z (Figures 2A,B). The weekly changes in abdominal circumference showed the same trend as that of body weight, with ECD treatment at 7 weeks of age significantly reducing the enlarged abdominal circumference (Figure 2C), which illustrated that ECD had intervention effects on abdominal obesity. However, the intervention effect of ECD was not realized through the control of food intake (Figure 2D).

Insulin sensitivity was evaluated by ITT at 9 weeks of age. The results revealed a higher blood glucose level at each time point and AUC in group Z, while ECD treatment effectively improved insulin sensitivity (Figures 2E,F). Additionally, fasting serum insulin levels and HOMA-IR indexes increased significantly in group Z, while ECD treatment attenuated IR of ZDF rats (Figures 2G,H).

The fat body ratio and blood lipids were standardized at the end of the experiment. The results showed an obvious increase of fat body ratio and various indexes of blood lipids in group Z. ECD markedly reduced epididymal and total fat body ratio, serum LDL-C, and TG (Figures 2I,J), indicating that ECD could regulate abnormal lipid metabolism *in vivo*. These data illustrated that ECD could effectively prevent and treat obesity and improve IR and lipid metabolism disorders in ZDF rats as expected.

The dose used in group EC-M is a clinically effective dose, which had basically same effect while less negative impact on liver and kidney function of rats compared with group EC-H (mainly manifested in significantly elevated ALT and more notably higher Cr level in group EC-H, as shown in Supplementary Figure 2). Therefore, EC-M was taken as the representative of treatment groups for subsequent studies.

### ECD Improved Insulin Signal Transduction and Decreased Lipolysis in WAT of ZDF Rats

**FIGURE 3:**
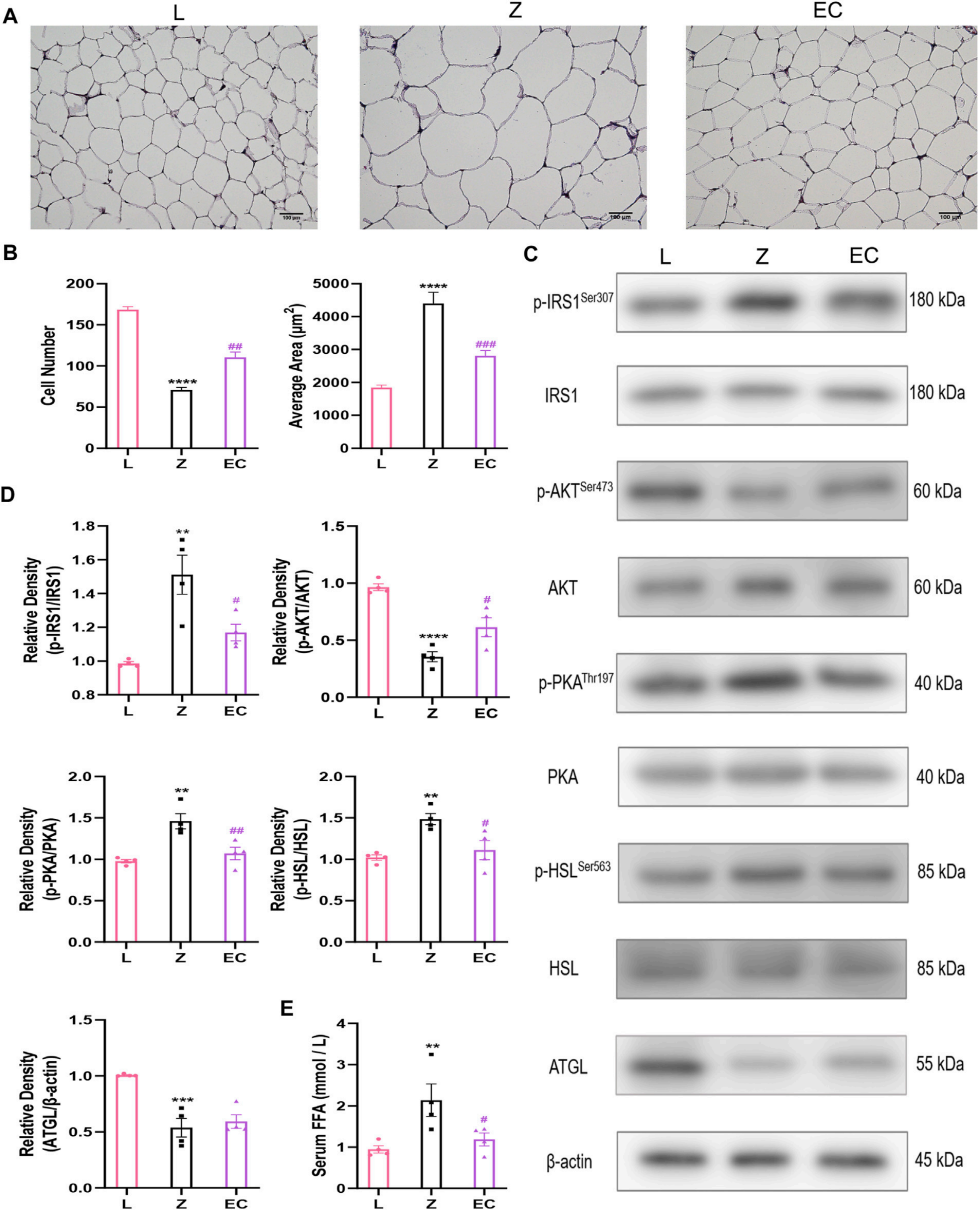
ECD improved insulin signal transduction and decreased lipolysis in WAT of ZDF rats. **(A)** Representative image (bar: 100 µM) and **(B)** number and average area of adipocytes of WAT HE staining (*n* = 3. Z vs. L, ****p < 0.0001; EC vs. Z, ^##^ *p* < 0.01, ^###^ *p* < 0.001). **(C)** Representative bands and (**D**) relative protein expression of p-IRS1/IRS1, p-AKT/AKT, p-PKA/PKA, p-HSL/HSL, and ATGL. **(E)** Serum FFA concentration (*n* = 4. Z vs. L, **p < 0.01, ***p < 0.001, ****p < 0.0001; EC vs. Z, ^#^ *p <* 0.05, ^##^ *p <* 0.01). (B), (D), and (E) were analyzed by one-way ANOVA.

WAT stores TG as an energy reserve and provides energy to tissues in the form of FFA. In pathological conditions, excessive lipolysis is a characteristic pathological change of obesity. At the histological level, HE staining results showed that the adipocytes in group L were uniform in size, clear in boundary and tightly arranged. However, in the same field of vision, the number of adipocytes decreased, the diameter and cross-sectional area of adipocytes increased in group Z, while ECD treatment increased the number of adipocytes, reduced the area of adipocytes tended to be normal (Figures 3A,B). Insulin has an important regulatory effect on lipolysis. It activates insulin signaling by binding to receptors on adipocytes and regulates downstream PKA activity. HSL, a key enzyme in the process of hydrolyzing diacylglycerol into glycerol and FFA, is an important target for PKA control. The expressions of p-IRS1/IRS1, p-AKT/AKT, p-PKA/PKA, and p-HSL/HSL in the epididymal WAT of ZDF rats were determined by western blotting to explore the underlying effect of ECD on lipolysis. The results showed that there were no significant changes in total protein levels of IRS1, AKT, PKA, or HSL levels in the three groups. However, we observed the differences in phosphorylation with a significant increase in p-IRS1/IRS1, p-PKA/PKA, and p-HSL/HSL levels and downregulation of the level of p-AKT/AKT in group Z. Compared with group Z, ECD could regulate the phosphorylation status of these molecules in the opposite direction, thereby improve significantly the activity. ATGL is the rate-limiting enzyme which decomposes triacylglycerols to diacylglycerol, which provides substrate for HSL. Contrary to HSL, its activity does not seem to be regulated by phosphorylation (Zimmermann et al., 2004). Therefore, the expression of ATGL protein was also measured. Compared with group L, ATGL protein content in group Z was notably reduced, but ECD did not modify it. These results suggested that there might be abnormal lipolysis in WAT of ZDF rats, and the effect of ECD on the lipolysis relied more on the improvement of IRS1/AKT/PKA/HSL signaling pathway rather than on a direct regulation of HSL or ATGL (Figures 3C,D). The regulation of insulin on ATGL is not mediated by AKT (Yin et al., 2019) or PKA (Zimmermann et al., 2004), which may explain why ECD had no effect on ATGL. The level of lipolysis affects the content of FFA in the circulation. Thus, we compared the concentrations of fasting serum FFA in three groups to confirm the effect of ECD on lipolytic function of ZDF rats. Compared with group L, the FFA concentration of group Z increased, while ECD treatment significantly reduced the FFA concentration of ZDF rats (Figure 3E). The above results indicated that there were abnormal cell morphology and excessive lipolysis in WAT of ZDF rats, and ECD could possess protective effect on the morphology of adipocytes and reduce the release of FFA from excessive lipolysis of WAT by interfering with insulin signal transduction, which might be related to the improvement of IRS1/AKT/PKA/HSL signaling pathway.

### ECD Modulated the Overall Structure and Composition of Gut Microbiota in ZDF Rats

To explore whether the biological effects of ECD were related to changes in gut microbiota, an important target for the development of obesity, fecal samples from rats at 5 and 9 weeks of age were collected and the 16S-V3V4 regions of the gut microbiota were pair-end sequenced using the Illumina high-throughput sequencing platform. A total of 1,610,611 sequences were gathered after denoising, and 1,113,709 high-quality sequences were obtained after quality control from 44 samples. The 17,692 sequence abundances of each sample ensured that all samples were analyzed at the same level of sequencing depth after leveling.

**FIGURE 4:**
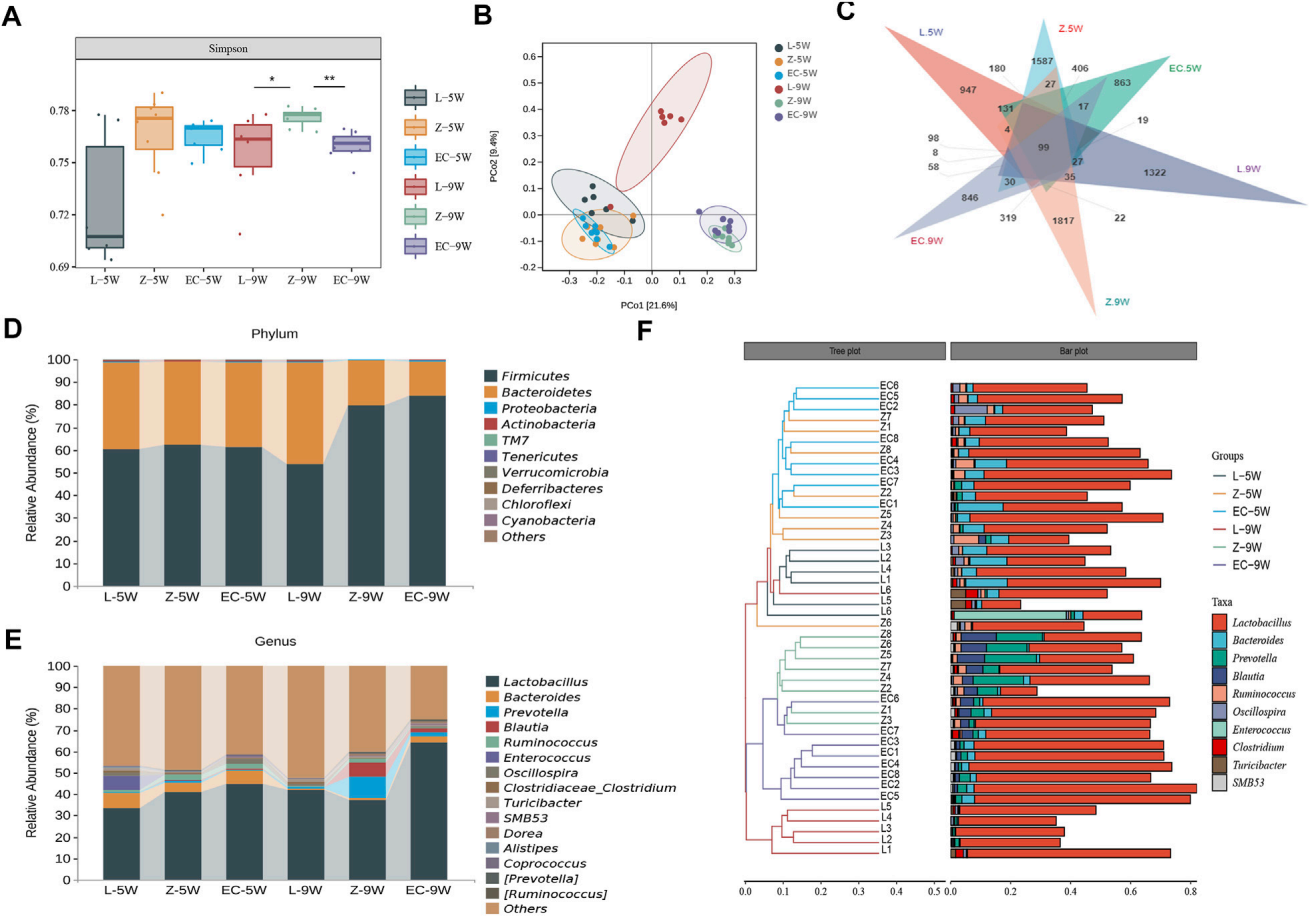
ECD modulated the overall structure and composition of gut microbiota in ZDF rats. Gut microbiota among groups at 5 and 9 weeks of age. **(A)** Simpson diversity. The Kruskal-Wallis rank sum test and Dunnett’s test were used as *post hoc* tests to verify the significance of the difference. ^*^ *p* < 0.05, ^**^ *p* < 0.01. **(B)** PCoA based on unweighted UniFrac distance. The ellipse confidence was 0.95. **(C)** ASV Venn diagram. **(D, E)** Gut microbiota composition at phylum and genus levels. **(F)** Hierarchical clustering analysis at the genus level. The left panel is a hierarchical clustering tree diagram and the right is a stacked column chart of genera.

We first assessed the changes in the structure of gut microbiota of rats. At 5 weeks of age, the Simpson index of ZDF rats was no different from that of the control. While at 9 weeks of age, the Simpson index of group Z was significantly higher than that of L, ECD intervention significantly reduced this index and changed the alpha diversity of gut microbiota in ZDF rats (Figure 4A). PCoA based on unweighted UniFrac distance (Figure 4B) illustrated that the bacterial structure of groups Z and L were separated significantly at 5 weeks of age. With the development of obesity, the bacterial structure of group Z changed (Z-5W vs. Z-9W: *R* ^2^ = 0.322,165, *p* = 0.002), and the difference between groups Z and L was more obvious at 9 weeks of age (L-5W vs. Z-5W: *R* ^2^ = 0.127,367, *p* = 0.002 < L-9W vs. Z-9W: *R* ^2^ = 0.321,536, *p* = 0.001). ECD also gradually changed the bacterial structure of ZDF rats (EC-5W vs. EC-9W: *R* ^2^ = 0.361,867, *p* = 0.002). At 9 weeks, the bacterial structures of EC and Z groups could be distinguished significantly (Z-5W vs. EC-5W: *R* ^2^ = 0.061437, *p* = 0.701 < Z-9W vs. EC-9W: *R* ^2^ = 0.238,092, *p* = 0.001).

We further observed the changes in the composition of gut microbiota of rats. We found that from 5 to 9 weeks of age, the shared ASVs between groups L and Z decreased from 890 to 312, and those between groups Z and EC decreased from 1,237 to 689 (Figure 4C), indicating that both the development of obesity and the intervention of ECD might cause some changes in the composition of rat gut microbiota. The top 10 phyla and top 15 genera in relative abundance of fecal microbiota in each group of rats at 5 and 9 weeks old are shown in Figures 4D,E, respectively. *Firmicutes* and *Bacteroidetes* were the two main phyla, followed by *Proteobacteria* and *Actinobacteria*, which was similar to the situation of human gut microbiota. At the genus level, *Lactobacillus* was the dominant genus in all stages of rats in each group. Hierarchical clustering analysis of the top 10 abundant genera of gut microbiota of each group at the two stages showed that the microbial composition of the EC group was similar to that of group Z at 5 weeks of age, while at 9 weeks of age, the microbial composition of the EC group was more similar to that of group L due to the intervention of ECD (Figure 4F). These results indicated that ECD gradually regulated the overall structure and genus composition of gut microbiota in ZDF rats.

### ECD Regulated the Abundance of Biomarkers at the Genus Level of Gut Microbiota in ZDF Rats

**FIGURE 5:**
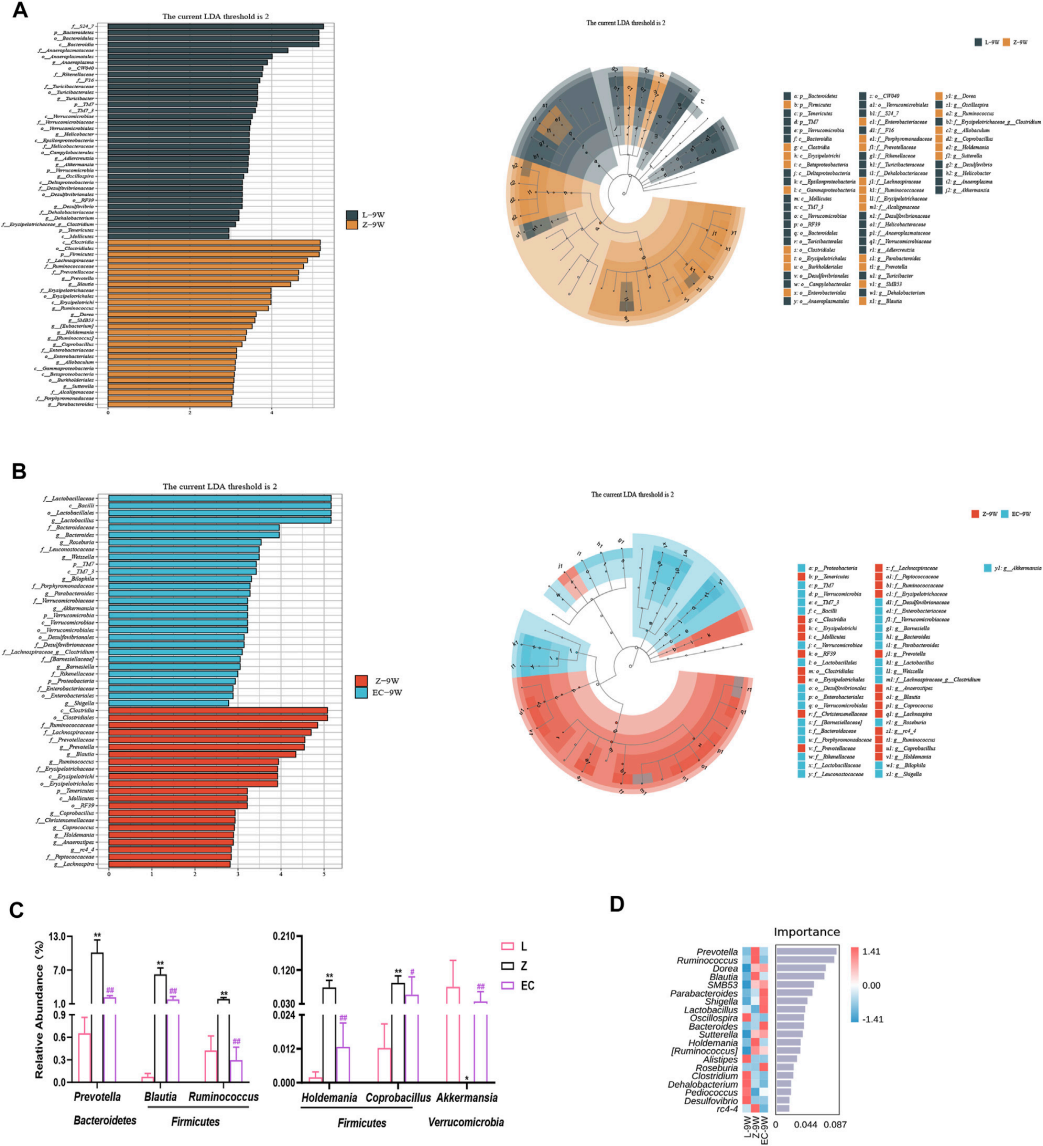
ECD regulated the abundance of biomarkers at the genus level of gut microbiota in ZDF rats. All three groups were at 9 weeks of age. **(A)** LDA value distribution histogram and cladogram of biomarkers between groups L and Z. (**B**) LDA value distribution histogram and cladogram of biomarkers between groups Z and EC. LDA score threshold >2 in (A) and (B). **(C)** Relative abundances of *Prevotella*, *Blautia*, *Ruminococcus*, *Holdemania*, *Coprobacillus*, and *Akkermansia* among three groups. The *p*-value was determined by LEfSe analysis. **(D)** Random forest analysis of differential gut microbiota. The intensity of colors represents the abundance distribution of gut microbiota in each sample (red, the corresponding abundance was higher; blue, the corresponding abundance was lower).

To detect biomarkers at the genus level, we compared horizontally the composition of gut microbiota among three groups at 5 and 9 weeks of age, and compared vertically the changes of gut microbiota of each group from 5 to 9 weeks of age. The gut microbiota changed significantly with the development of obesity and ECD treatment were explored. We found that the differences of gut microbiota at genus level were not significant at 5 weeks of age. However, from 5 to 9 weeks, the promotion of nine genera (*Prevotella*, *Blautia*, *Dorea*, *SMB53*, *Allobaculum*, *Coprobacillus*, [*Ruminococcus*], *Holdemania*, and *Sutterella*) and the reduction of five genera (*Akkermansia, Oscillospira*, *Adlercreutzia*, *Dehalobacterium*, and *f_Erysipelotrichaceae_g_Clostridium*) were established during the development of obesity, which had a significant difference in group Z comparison to L at 9 weeks of age, implying the potential relevance of these genera to obesity progression (Supplementary Table 3). At the same time, ECD treatment gradually changed the relative abundance of four genera mentioned above, including decreasing *Prevotella*, *Blautia, Coprobacillus* and *Holdemania*, and increasing *Akkermansia*. In addition, ECD also gradually reduced the amount of *Ruminococcus*. At 9 weeks of age, the relative abundance of these genera in group EC were markedly different from group Z and tended to a normal level, which are the bacterial targets of ECD. LDA value distribution histogram and corresponding cladogram were used to show microbiota and their taxonomic hierarchies with significant differences between groups at 9 weeks of age (Figures 5A,B). The relative abundances of ECD intervention biomarkers are shown in Figure 5C, and their LDA and *p* values are shown in Supplementary Table 4. The importance order of these genera is shown by random forest analysis. In particular, *Prevotella*, *Ruminococcus*, *Blautia* and *Holdemania* have a greater impact on the formation of differences among groups (Figure 5D).

### ECD Regulated the Function of Gut Microbiota in ZDF Rats

**FIGURE 6:**
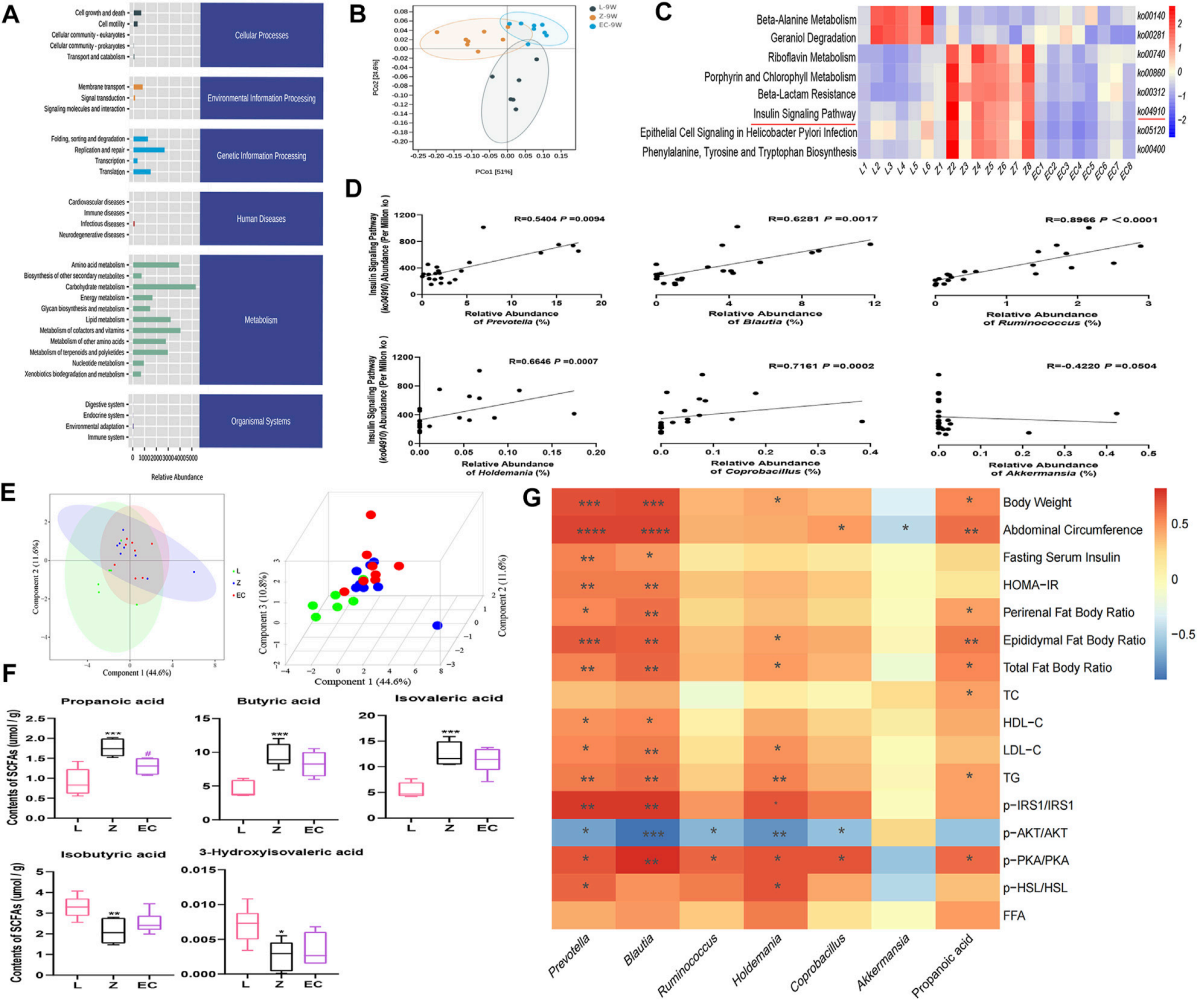
ECD regulated the function of gut microbiota in ZDF rats. All three groups were at 9 weeks of age. **(A)** The abundance of KEGG functional pathways at the secondary classification level of gut microbiota. **(B)** PCoA of gut microbiota functional units based on Bray-Curtis similarity. The ellipse confidence was 0.95. **(C)** Different metabolic pathways of gut microbiota. The intensity of colors represents the degree of association between gut microbiota and signaling pathways in each sample (red, strong correlation; blue, low correlation). **(D)** Correlation analysis between *Prevotella*, *Blautia*, *Ruminococcus*, *Holdemania*, *Coprobacillus*, *Akkermansia*, and the insulin signaling pathway (*ko04910*). **(E)** 2D and 3D PLS-DA of SCFAs. **(F)** Contents of propionic, butyric, isovaleric, isobutyric, and 3-hydroxyisovaleric acid in cecal contents. The differences were analyzed by one-way ANOVA (Z vs. L, ^*^ *p* < 0.05, ^**^ *p* < 0.01, ^***^ *p* < 0.001; EC vs. Z, ^#^ *p* < 0.05). **(G)** Correlation between microbial and SCFA biomarkers and host phenotype. The panel shows the associations of six different genera and propionic acid with obesity, IR and lipid metabolism disorder phenotypes, and expression of the IRS1/AKT/PKA/HSL signaling pathway. Color intensity represents the degree of association (red, positive correlation; blue, negative correlation). ^*^ *p* < 0.05, ^**^ *p* < 0.01, ^***^ *p* < 0.001, ^****^ *p* < 0.0001. Except (F) *n* = 5–8, the rest *n* = 6–8.

To observe whether the changes in the composition of gut microbiota further leads to functional changes, we further carried out the prediction of the function of microbiota, and detected the changes in the content of important microbiota metabolites, SCFAs. The function of gut microbiota in 9-week-old rats was mainly focused on genetic information processing and metabolism, especially energy metabolism and the metabolism of the three major energy substances, amino acids, carbohydrates, and lipid (Figure 6A). However, PCoA showed that there was a certain separation of microbial functions among the three groups in rats. In the PC1 dimension, the functional composition of the EC group was more similar to that of group L, with a contribution rate of 51% (Figure 6B). There were significant differences in eight signaling pathways, including the insulin signaling pathway (*ko04910*) (Figure 6C). Its abundance was significantly positively correlated with the relative abundance of *Prevotella*, *Blautia*, *Ruminococcus*, *Holdemania*, and *Coprobacillus* (Figure 6D), implying a potential role for these ECD intervention biomarkers. The metabolites secreted, modified, and degraded by gut microbiota are important mediators of the host-microbiota dialogue, which participate in the regulation of host metabolism. SCFAs are metabolites that have a high concentration in the cecum, and mainly include acetic acid, propionic acid, and butyric acid. The metabolism of SCFAs in the cecum of groups at 9 weeks of age was analyzed. The results showed that ZDF and ZL rats were separated in their composition of SCFAs (Figure 6E). Compared with group L, there were significant changes in the contents of five SCFAs in group Z, among which propionic, butyric, and isovaleric acid were notably increased, while isobutyric and 3-hydroxyisovaleric acid were obviously reduced. ECD treatment significantly reduced propionic acid and tended to reduce butyric and isovaleric acid while raising isobutyric and 3-hydroxyisovaleric acid in the cecum of rats (Figure 6F). *Prevotella* (De Vadder et al., 2016), *Blautia* (Reichardt et al., 2014) and *Ruminococcus* (Krautkramer et al., 2020) strains have been reported to produce propionic acid by fermentation. Therefore, ECD might reduce the abundance of these bacteria to reduce the content of propionic acid. Acetic acid is the fermentation product of most intestinal bacteria. Butyric acid- and propionic acid-producing bacteria were almost different, which explains why there were no differences in acetic acid among the groups or a significant change in the content of butyric acid after the intervention.

ECD significantly improved IR and lipid metabolism disorders, especially in WAT of ZDF rats. Therefore, we analyzed the correlation between the six core genera and propionic acid, which changed after ECD intervention, and host phenotype (Figure 6G). We observed that *Prevotella* and *Blautia* were not only markedly correlated positively with obesity phenotypes such as body weight and abdominal circumference, but also with pathological manifestations such as IR and abnormal lipid metabolism, while *Holdemania* was mainly associated positively with lipid metabolism disorder-related indexes. *Prevotella*, *Blautia, Ruminococcus, Holdemania,* and *Coprobacillus* also showed different degrees of correlation with the expression of the IRS1/AKT/PKA/HSL signaling pathway in WAT. In addition, the content of the gut microbiota metabolite propionic acid was significantly positively correlated with body weight, abdominal circumference, and phenotypes related to lipid metabolism disorders. These results revealed a potentially close relationship between the host phenotype and biomarkers, especially *Prevotella*, *Blautia*, *Holdemania*, and propionic acid. These might be important targets for ECD to improve obesity, especially lipid metabolism disorders via gut microbiota. The regulation of the insulin signaling pathway might also play an important role.

## Discussion

In this study, we found for the first time that ECD changes the composition and function of gut microbiota in ZDF rats, which led them towards a healthier state. Interestingly, the changes in gut microbiota induced by ECD intervention were closely related to the improvement of IR and lipid metabolism disorders, especially in WAT, indicating that the beneficial effects of ECD on obesity, especially lipid metabolism disorders, were related to the regulation of gut microbiota in ZDF rats.

ZDF rats are characterized by obesity, IR, and hyperlipidemia due to mutations in the extracellular region of the leptin receptor (Habegger et al., 2014). Compared with traditional dietary model, this animal model has a shorter time interval and is stable, which makes it ideal to study obesity. We found that ECD treatment could help the negative effects of obesity, including weight loss, improvements in IR, and the regulation of dyslipidemia, which is consistent with previous studies (Gao et al., 2015; Zhang et al., 2017; Ding et al., 2018; Zhang et al., 2020b; Lee et al., 2020) and illustrates that ECD has a regulatory effect on obesity caused by genetic factors. However, the regulatory effects of ECD on TC and HDL-C in blood lipids are not currently consistent, which might be related to the different models and drug concentrations.

Previous studies have investigated the biological effects by which ECD modulates metabolism. ECD can promote the expression of CDKAL1 and improve the function of islet cells, thereby ameliorating insulin secretion (Gao et al., 2015). Moreover, the intervention effects by which ECD improves lipid metabolism include the inflammatory response (Lee et al., 2020) and lipid transport (Ding et al., 2018). The lipid metabolic balance is the result of a combination of lipogenesis and lipolysis. Studies have found that ECD upregulates the expression of peroxisome proliferator-activated receptor gamma (PPARγ) in visceral fat and skeletal muscle and lipoprotein lipase (LPL) in skeletal muscle (Zhang et al., 2020b), and reduces the lipid accumulation caused by IR by inhibiting the expression of IRS1 phosphorylation in the liver (Zhang et al., 2017). Adipose tissue is an important target organ for the treatment of obesity (Kusminski et al., 2016), as it expands in obese individuals. Due to homeostatic regulation and continuous low-level inflammation (Shiau et al., 2019), excessive fat leads to lipolysis, resulting in FFA releases to the circulation and liver, skeletal muscle, pancreas, and other tissues, which leads to lipid toxicity and IR throughout the body. In particular, visceral adipocyte hypertrophy results in decreased insulin sensitivity, a weakened insulin anti-lipolytic effect, and enhanced lipolytic activity in adipocytes (Roden and Shulman, 2019). Studies have found that the anti-lipolytic effect of insulin can be adjusted through the AKT/PKA/HSL signaling pathway (Yin et al., 2019), and inhibiting excessive lipolysis of adipose tissue (Park et al., 2020) is an important way to treat metabolic diseases. Consistent with previous results, we found that adipocytes expanded, IRS1 phosphorylation increased, AKT activity decreased, and insulin signal transduction was impaired, while PKA-mediated HSL activity was upregulated and the ability to release FFA into the circulation was enhanced in the WAT of ZDF rats. ECD not only changed the histological morphology, but also improved lipolysis in WAT by modulating the IRS1/AKT/PKA/HSL signaling pathway and reverting it to normal. Under the condition of basically the same food intake and calories, ECD changed the weight of ZDF rats, which might be related to the increase of energy consumption. Both previous studies and our studies have shown that ECD could improve the metabolic regulation of adipose tissue, which might be accompanied by changes in the function of mitochondria in adipose tissue, because the location (Brestoff et al., 2020) and activity (Joffin et al., 2021) of mitochondria in adipose tissue play a key role in the homeostasis of lipid metabolism.

Host genetics affect the composition of gut microbiota (Goodrich et al., 2014), and gut microbiota in turn regulate host energy homeostasis and glucose and lipid metabolism (Tremaroli and Backhed, 2012). In addition, environmental factors also importantly affect the gut microbiota (Rothschild et al., 2018). Many studies have revealed a close underlying connection between changes in gut microbiota and the occurrence and development of obesity. Targeting gut microbiota could improve insulin sensitivity (Udayappan et al., 2016), thereby regulating insulin-mediated lipid metabolism in adipocytes (Kimura et al., 2013) and improving host obesity. In our previous work, we observed the effects of fecal microbiota transplantation on the progression of obesity-susceptible diabetic mellitus (Zhang L. et al., 2020) and dynamic changes in fecal microbiota in the diabetic mellitus stage of ZDF rats (Zhou et al., 2019). We revealed a potential role for the gut microbial structure and composition in the disease progression of ZDF rats. At the same time, we found that the traditional Chinese medicine formula, ECD could delay the development of obesity in ZDF rats. Based on this, here we investigated the intervention effect of ECD on gut microbiota in the obesity stage of ZDF rats. We found that ECD reversed the changed diversity, adjusted the overall structure, and shifted the composition of gut microbiota at the genus level to render them normal during the development of obesity, especially the relative abundances of *Prevotella*, *Blautia*, *Ruminococcus*, *Holdemania*, *Coprobacillus*, and *Akkermansia* in ZDF rats. Current studies indicate that the association of *Prevotella* and *Blautia* with host health or disease status is controversial. Some researchers believe that increased *Prevotella* abundance can promote glycogen storage (Kovatcheva-Datchary et al., 2015) and produce succinic acid to activate intestinal gluconeogenesis, which is related to the improvement of glucose metabolism and insulin tolerance (De Vadder et al., 2016). However, some studies have found that *Prevotella* can participate in the biosynthesis of branched-chain amino acids, which is an important risk factor for the decreased insulin sensitivity, glucose tolerance, and the occurrence of type 2 diabetes (De Filippis et al., 2019). In addition, high levels of *Prevotella* can activate immune and stromal cells to release more inflammatory mediators, promote chronic inflammation (Larsen, 2017), and participate in the disease process. In obese (Si et al., 2017) and non-alcoholic fatty liver disease (Zhu et al., 2013; Schwimmer et al., 2019) individuals, *Prevotella* is significantly enriched. We found that ECD intervention reduced the elevated *Prevotella* in ZDF rats, and in addition to insulin resistance, *Prevotella* abundance was markedly positively correlated with lipid metabolism disorders, while previous studies focused on carbohydrate and amino acid metabolic pathways (Petersen et al., 2017). *Blautia* is an important SCFA producing bacteria (Liu et al., 2015), with anti-inflammatory effects (Benítez-Páez et al., 2020) that aid in the recovery of intestinal mucosal damage (Zhou et al., 2017); it is inversely associated with visceral fat content (Ozato et al., 2019) and plays a beneficial therapeutic role in metabolic disorders (Rodriguez et al., 2020). However, some studies have suggested that higher *Blautia* is associated with increased intestinal permeability (Leclercq et al., 2014), and its abundance is positively correlated with metabolic diseases and related to cardiovascular disease predictors such as plasma glutamate and branched-chain amino acids (Ottosson et al., 2018). In metabolic diseases such as obesity (Stanislawski et al., 2017), diabetes (Egshatyan et al., 2016; Wei et al., 2018), and nonalcoholic steatohepatitis (Del Chierico et al., 2017), the abundance of *Blautia* is increased. This might be related to the decrease in the abundance of other SCFA producing bacteria (Becker et al., 2011), or the result of inflammatory responses in different disease stages (Tuovinen et al., 2013). Our results indicated that ECD reduced *Blautia*, which was remarkably enriched in ZDF rats, and its abundance was significantly positively correlated with the negative effects of insulin sensitivity and lipid metabolism. *Ruminococcus* can degrade resistant starches (Ze et al., 2012), thereby increasing intestinal energy absorption, which promotes weight gain in individuals (Cotillard et al., 2013). It also affects intestinal health by promoting oxidative stress (Hall et al., 2017) and inflammatory responses (Rajilić-Stojanović et al., 2015; van den Munckhof et al., 2018), and is considered to be related to negative human health consequences (Hills et al., 2019). ECD reduced the relative abundance of this genus. Members of the *Erysipelotrichaceae* family are closely related to clinical indicators of impaired glucose and lipid metabolism and are important targets of metabolic diseases (Kaakoush, 2015; Lippert et al., 2017). Both *Holdemania* and *Coprobacillus* are members of the *Erysipelotrichaceae* family. *Holdemania* is related to the occurrence of inflammatory reaction (Barandouzi et al., 2020; Jang et al., 2020), elevated in patients with type 1 diabetes (Biassoni et al., 2020), and is considered to be a predictor of hypertension (Hsu et al., 2020). *Coprobacillus* is an important butyric acid producer and can be cross-fed with *Anaerostipes, Roseburia*, and *Bifidobacterium* to maintain butyric acid concentrations in the colon (Muthuramalingam et al., 2020). It affects intestinal function and mediates related intestinal diseases (Kassinen et al., 2007) through the inflammatory response (Shi et al., 2018; Seo et al., 2019). Its abundance is also positively correlated with the expression of immune function related genes (Elderman et al., 2018). Through the influence of lipid metabolism (Kim et al., 2018), its abundance in the intestines of obese animals and humans is increased (Wang et al., 2018; Terzo et al., 2020). ECD effectively reduced the relative abundance of these two genera of *Erysipelotrichaceae* in ZDF rats, and we found that the relative abundance of *Holdemania* was significantly and positively correlated with abnormal lipid metabolism. *Akkermansia* is currently one of the most widely studied probiotics, and it might be suitable for treating metabolic syndrome. It can improve metabolic disorders in obese animals and humans, including decreased insulin sensitivity and glucose and lipid metabolism disorders (Anhê et al., 2015; Dao et al., 2016; Depommier et al., 2019). It restores intestinal barrier function (Desai et al., 2016) through the immunomodulatory effect of cell membrane protein AMUC-1100 binding to toll-like receptor 2 (Plovier et al., 2017) reducing macrophage infiltration, proinflammatory cytokines, and chemokine expression, therefore reducing the risk of cardiovascular disease (Li et al., 2016). ECD increased the relative abundance of *Akkermansia* that was decreased in ZDF rats. The inconsistency of current research results is not only related to differences in disease states, animal models, interventions, diets, etc., but also indicates that effects of microbiota cannot be generalized simply as beneficial or harmful. Differences at the species level might lead to different results, and disease phenotypes are often only related to a small number of strains (Truong et al., 2017). Therefore, it is necessary to further explore the changes of specific strains under each genus in future research.

Gut microbiota is an important endogenous factor in regulating WAT browning (Li et al., 2017) and brown adipose tissue activity (Quan et al., 2020), and it can regulate WAT inflammation (Virtue et al., 2019) and affect WAT function. Studies have showed that intestinal barrier injury in obese individuals can lead to the translocation of intestinal flora or flora components (Anhê et al., 2020), and the number of bacteria in adipose tissue is related to immune cell infiltration, inflammation, and metabolic indicators, which affect the metabolic health of obese individuals (Massier et al., 2020). Treatment with obesity-related harmful strains increases the hypertrophy of adipocytes in obese mice, resulting in decreased insulin sensitivity and increased lipolysis in adipose tissue (Keskitalo et al., 2018). We found that the relative abundances of *Prevotella*, *Blautia*, and *Holdemania* were not only clearly positively correlated with the host phenotype, but also with the expression of the IRS1/AKT/PKA/HSL signaling pathway in WAT, suggesting that *Prevotella*, *Blautia*, and *Holdemania* might be important targets for ECD to enhance insulin sensitivity, thereby reducing excessive lipolysis in WAT of ZDF rats. However, the specific mechanism is still unclear, which is a direction worthy of attention in future research.

This study is the first to examine gut microbiota targets of ECD intervention. In addition to gut microbiota, their metabolite SCFAs may be an important pathway for exerting their metabolic effects (De Vadder et al., 2014). Although SCFAs are related to metabolism, the role of SCFAs in energy homeostasis is ambiguous at present (Canfora et al., 2015). Some animal and human studies have shown that obesity is associated with high levels of SCFAs (Freeland and Wolever, 2010; Kim et al., 2019). Gut microbiota ferment undigested carbohydrates (such as resistant starch and dietary fiber) and proteins in the small intestine to produce SCFAs, which increases energy absorption and then *de novo* synthesis of lipids and glucose in the whole body, providing about 10% of an individual’s energy requirements, potentially leading to obesity (Turnbaugh et al., 2006). Consistently, propionic and butyric acid, the two most important SCFAs, increased significantly in the cecal contents of 9-week-old ZDF rats. This change might be the result of an increase in intestinal bacteria producing these two SCFAs or a decrease in bacteria utilizing them in the intestinal tract of ZDF rats. The changes may also be related to the fermentation or utilization rates of different gut microbiota, microbial cross-feeding, mucosal absorption and transport rate and other complex factors (Schwiertz et al., 2010; Fernandes et al., 2014). Studies have shown that butyric acid is the main energy source for intestinal epithelial cells and can increase lipid synthesis (Birt et al., 2013). The presence of propionic acid in feces is related to increased risk of type 2 diabetes (Sanna et al., 2019). Furthermore, both propionic and butyric acid can stimulate lipolysis in adipocytes (Rumberger et al., 2014). We found that the content of propionic acid was significantly positively correlated with the phenotypes of obesity and lipid metabolism disorders. Decreased propionic acid content could be used as an independent predictor of the improvement of insulin sensitivity (Tirosh et al., 2019). ECD administration reduced the concentration of propionic acid in ZDF rats, possibly by adjusting the gut microbiota to change the content of fermentation products. Studies have shown that *Blautia* strains ferment the deoxy sugars rhamnose and fucose to form propionic acid through the propylene glycol pathway (Reichardt et al., 2014). *Prevotella* (De Vadder et al., 2016) and *Ruminococcus* (Krautkramer et al., 2020) produce succinate, an intermediate product of propionic acid, through the succinate pathway. Therefore, propionic acid might be an important medium for gut microbiota of ECD intervention and a subject for future research. Different SCFAs might exert their biological effects through synergy and antagonism (Li et al., 2020). In addition, the content of SCFAs in different intestinal segments is different (Cummings et al., 1987), and SCFAs in circulation are more closely related to peripheral insulin sensitivity, systemic lipolysis, and metabolic health (Müller et al., 2019). Therefore, the regulatory effect of ECD on SCFAs still needs to be further explored.

In conclusion, we found that ECD could regulate lipid metabolism, improve lipolysis in WAT, and modulate the composition and function of gut microbiota in ZDF rats. There was a significant correlation between biomarkers and host phenotype, suggesting that the beneficial effects of ECD on obesity, especially lipid metabolism disorders, were related to the modulation of gut microbiota. The limitations of this research were that, first of all, isoflurane anesthesia may aggravate the pre-existing IR (Fang et al., 2020), thereby affecting the judgment of the degree of IR in ZDF rats. Secondly, genetic levels and even more molecular experiments may be required to confirm the complex crosstalk among molecules for the changes in the lipolytic function of WAT. Moreover, the dietary factors cannot be ignored. The dietary components of ZL and ZDF rats were different (Supplementary Table 1), and the food intake of ZDF rats was much higher than that of ZL rats (Figure 2D), which led to different types and amounts of substrates fermented by gut microbiota, resulting in metabolic differences (Makki et al., 2018). Finally, the causal relationship between the regulation of gut microbiota by ECD and the improvement of lipid metabolism remains to be further explored.

## Conclusion

We found that ECD delayed the development of obesity, inhibited excessive lipolysis by improving the activity of the IRS1/AKT/PKA/HSL signaling pathway in WAT of ZDF rats. In addition, ECD had an impact on the composition and function of obesity-related gut microbiota, reduced the content of *Prevotella, Blautia*, and *Holdemania*, and the metabolite propionic acid. These biomarkers were significantly positively correlated with host obesity phenotype, especially lipid metabolism disorders. This study provides new insights into the role of ECD in improving obesity and regulating lipid metabolism disorders via gut microbiota and helps to further clarify the mechanism of ECD in the treatment of obesity.

## Data Availability

The raw sequences of Miseq sequences from 44 fecal samples of rats have been submitted to NCBI Project under accession number PRJNA686642 with NCBI Sequence Read Archive under accession number SRP298569.

## Footnotes

1 World Health Organization. (2020). Obesity and overweight. https://www.who.int/news-room/fact-sheets/detail/obesity-and-overweight. [Accessed December 20, 2020].

## Glossary

AKT: protein kinase B
ALT: alanine aminotransferase
ANOVA: analysis of variance
AST: aspartate aminotransferase
ASVs: amplitude sequence variables
ATGL: adipose triglyceride lipase
AUC: area under the curve
BUN: blood urea nitrogen
Cr: creatinine
ECD: Erchen Decoction
FFA: free fatty acids
HDL-C: high-density lipoprotein cholesterol
HE: hematoxylin-eosin
HOMA-IR: homeostasis model assessment-insulin resistance
HPLC: high performance liquid chromatography
HSL: hormone-sensitive triglyceride lipase
IR: insulin resistance
IRS1: insulin receptor substrate 1
ITT: insulin tolerance test
LDA: linear discriminant analysis
LDL-C: low-density lipoprotein cholesterol
LEfSe: LDA effect size
PCoA: principal coordinates analysis
PKA: protein kinase A
PLS-DA: partial least squares discrimination analysis
SCFAs: short-chain fatty acids
SEM: standard error of the mean
TC: total cholesterol
TG: triglycerides
UHPLC-ESI-Q-TOF-MS: ultraperformance liquid chromatography-electrospray ionization-quadrupole-time of flight-mass spectrometry
UPLC-MS/MS: ultraperformance liquid chromatography-tandem mass spectrometry
WAT: white adipose tissue
ZDF rats: Zucker diabetic fatty rats
ZL rats: Zucker lean rats

## References

[B1] Anhê F. F. Jensen B. A. H. Varin T. V. Servant F. Van Blerk S. Richard D. (2020). Type 2 Diabetes Influences Bacterial Tissue Compartmentalisation in Human Obesity. Nat. Metab. 2 (3), 233–242. 10.1038/s42255-020-0178-9 32694777

[B2] Anhê F. F. Roy D. Pilon G. Dudonné S. Matamoros S. Varin T. V. (2015). A Polyphenol-Rich cranberry Extract Protects from Diet-Induced Obesity, Insulin Resistance and Intestinal Inflammation in Association with increasedAkkermansiaspp. Population in the Gut Microbiota of Mice. Gut 64 (6), 872–883. 10.1136/gutjnl-2014-307142 25080446

[B3] Barandouzi Z. A. Starkweather A. R. Henderson W. A. Gyamfi A. Cong X. S. (2020). Altered Composition of Gut Microbiota in Depression: A Systematic Review. Front. Psychiatry 11, 541. 10.3389/fpsyt.2020.00541 32587537 PMC7299157

[B4] Becker N. Kunath J. Loh G. Blaut M. (2011). Human Intestinal Microbiota: Characterization of a Simplified and Stable Gnotobiotic Rat Model. Gut Microbes 2 (1), 25–33. 10.4161/gmic.2.1.14651 21637015

[B5] Benítez-Páez A. Gómez del Pugar E. M. López-Almela I. Moya-Pérez Á. Codoñer-Franch P. Sanz Y. (2020). Depletion of Blautia Species in the Microbiota of Obese Children Relates to Intestinal Inflammation and Metabolic Phenotype Worsening. mSystems 5 (2), e00857–19. 10.1128/mSystems.00857-19 32209719 PMC7093825

[B6] Biassoni R. Di Marco E. Squillario M. Barla A. Piccolo G. Ugolotti E. (2020). Gut Microbiota in T1DM-Onset Pediatric Patients: Machine-Learning Algorithms to Classify Microorganisms as Disease Linked. J. Clin. Endocrinol. Metab. 105 (9), e3114–e3126. 10.1210/clinem/dgaa407 32692360

[B7] Birt D. F. Boylston T. Hendrich S. Jane J.-L. Hollis J. Li L. (2013). Resistant Starch: Promise for Improving Human Health. Adv. Nutr. 4 (6), 587–601. 10.3945/an.113.004325 24228189 PMC3823506

[B8] Brestoff J. R. Wilen C. B. Moley J. R. Li Y. Zou W. Malvin N. P. (2021). Intercellular Mitochondria Transfer to Macrophages Regulates White Adipose Tissue Homeostasis and Is Impaired in Obesity. Cel Metab. 33 (2), 270–282. 10.1016/j.cmet.2020.11.008 PMC7858234 33278339

[B9] Callahan B. J. McMurdie P. J. Rosen M. J. Han A. W. Johnson A. J. A. Holmes S. P. (2016). DADA2: High-Resolution Sample Inference from Illumina Amplicon Data. Nat. Methods 13 (7), 581–583. 10.1038/nmeth.3869 27214047 PMC4927377

[B10] Canfora E. E. Jocken J. W. Blaak E. E. (2015). Short-chain Fatty Acids in Control of Body Weight and Insulin Sensitivity. Nat. Rev. Endocrinol. 11 (10), 577–591. 10.1038/nrendo.2015.128 26260141

[B11] Caprio S. Perry R. Kursawe R. (2017). Adolescent Obesity and Insulin Resistance: Roles of Ectopic Fat Accumulation and Adipose Inflammation. Gastroenterology 152 (7), 1638–1646. 10.1053/j.gastro.2016.12.051 28192105 PMC9390070

[B12] Chinese Pharmacopoeia Commission (2015). Pharmacopoeia of the People’s republic of China, Vol. I. China: China Medical Science Press.

[B13] Cotillard A. Kennedy S. P. Kong L. C. Prifti E. Pons N. Le Chatelier E. (2013). Dietary Intervention Impact on Gut Microbial Gene Richness. Nature 500 (7464), 585–588. 10.1038/nature12480 23985875

[B14] Cummings J. H. Pomare E. W. Branch W. J. Naylor C. P. Macfarlane G. T. (1987). Short Chain Fatty Acids in Human Large Intestine, portal, Hepatic and Venous Blood. Gut 28 (10), 1221–1227. 10.1136/gut.28.10.1221 3678950 PMC1433442

[B15] Czech M. P. (2017). Insulin Action and Resistance in Obesity and Type 2 Diabetes. Nat. Med. 23 (7), 804–814. 10.1038/nm.4350 28697184 PMC6048953

[B16] Dao M. C. Everard A. Aron-Wisnewsky J. Sokolovska N. Prifti E. Verger E. O. (2016). Akkermansia Muciniphilaand Improved Metabolic Health during a Dietary Intervention in Obesity: Relationship with Gut Microbiome Richness and Ecology. Gut 65 (3), 426–436. 10.1136/gutjnl-2014-308778 26100928

[B17] De Filippis F. Pasolli E. Tett A. Tarallo S. Naccarati A. De Angelis M. (2019). Distinct Genetic and Functional Traits of Human Intestinal Prevotella Copri Strains Are Associated with Different Habitual Diets. Cell Host & Microbe 25 (3), 444–453. 10.1016/j.chom.2019.01.004 30799264

[B18] De Vadder F. Kovatcheva-Datchary P. Goncalves D. Vinera J. Zitoun C. Duchampt A. (2014). Microbiota-generated Metabolites Promote Metabolic Benefits via Gut-Brain Neural Circuits. Cell 156 (1-2), 84–96. 10.1016/j.cell.2013.12.016 24412651

[B19] De Vadder F. Kovatcheva-Datchary P. Zitoun C. Duchampt A. Bäckhed F. Mithieux G. (2016). Microbiota-Produced Succinate Improves Glucose Homeostasis via Intestinal Gluconeogenesis. Cell Metab 24 (1), 151–157. 10.1016/j.cmet.2016.06.013 27411015

[B20] Del Chierico F. Nobili V. Vernocchi P. Russo A. De Stefanis C. Gnani D. (2017). Gut Microbiota Profiling of Pediatric Nonalcoholic Fatty Liver Disease and Obese Patients Unveiled by an Integrated Meta‐omics‐based Approach. Hepatology 65 (2), 451–464. 10.1002/hep.28572 27028797

[B21] Depommier C. Everard A. Druart C. Plovier H. Van Hul M. Vieira-Silva S. (2019). Supplementation with Akkermansia Muciniphila in Overweight and Obese Human Volunteers: a Proof-Of-Concept Exploratory Study. Nat. Med. 25 (7), 1096–1103. 10.1038/s41591-019-0495-2 31263284 PMC6699990

[B22] Desai M. S. Seekatz A. M. Koropatkin N. M. Kamada N. Hickey C. A. Wolter M. (2016). A Dietary Fiber-Deprived Gut Microbiota Degrades the Colonic Mucus Barrier and Enhances Pathogen Susceptibility. Cell 167 (5), 1339–1353. 10.1016/j.cell.2016.10.043 27863247 PMC5131798

[B23] Ding S. Kang J. Tong L. Lin Y. Liao L. Gao B. (2018). Erchen Decoction Ameliorates Lipid Metabolism by the Regulation of the Protein CAV-1 and the Receptors VLDLR, LDLR, ABCA1, and SRB1 in a High-Fat Diet Rat Model. Evidence-Based Complement. Altern. Med. 2018, 1–12. 10.1155/2018/5309490 PMC6196931 30402126

[B24] Egshatyan L. Kashtanova D. Popenko A. Tkacheva O. Tyakht A. Alexeev D. (2016). Gut Microbiota and Diet in Patients with Different Glucose Tolerance. Endocr. Connect. 5 (1), 1–9. 10.1530/EC-15-0094 26555712 PMC4674628

[B25] Elderman M. Hugenholtz F. Belzer C. Boekschoten M. van Beek A. de Haan B. (2018). Sex and Strain Dependent Differences in Mucosal Immunology and Microbiota Composition in Mice. Biol. Sex. Differ. 9 (1), 26. 10.1186/s13293-018-0186-6 29914546 PMC6006852

[B26] Fang X. Xia T. Xu F. Wu H. Ma Z. Zhao X. (2020). Isoflurane Aggravates Peripheral and central Insulin Resistance in High-Fat Diet/streptozocin-Induced Type 2 Diabetic Mice. Brain Res. 1727, 146511. 10.1016/j.brainres.2019.146511 31672472

[B27] Feng Y.-L. Cao G. Chen D.-Q. Vaziri N. D. Chen L. Zhang J. (2019). Microbiome-metabolomics Reveals Gut Microbiota Associated with Glycine-Conjugated Metabolites and Polyamine Metabolism in Chronic Kidney Disease. Cell. Mol. Life Sci. 76 (24), 4961–4978. 10.1007/s00018-019-03155-9 31147751 PMC11105293

[B28] Fernandes J. Su W. Rahat-Rozenbloom S. Wolever T. M. S. Comelli E. M. (2014). Adiposity, Gut Microbiota and Faecal Short Chain Fatty Acids Are Linked in Adult Humans. Nutr. Diabetes 4 (6), e121. 10.1038/nutd.2014.23 24979150 PMC4079931

[B29] Freeland K. R. Wolever T. M. S. (2010). Acute Effects of Intravenous and Rectal Acetate on Glucagon-like Peptide-1, Peptide YY, Ghrelin, Adiponectin and Tumour Necrosis Factor-α. Br. J. Nutr. 103 (3), 460–466. 10.1017/S0007114509991863 19818198

[B30] Frühbeck G. Méndez-Giménez L. Fernández-Formoso J.-A. Fernández S. Rodríguez A. (2014). Regulation of Adipocyte Lipolysis. Nutr. Res. Rev. 27 (1), 63–93. 10.1017/S095442241400002X 24872083

[B31] Gao B.-Z. Chen J.-C. Liao L.-H. Xu J.-Q. Lin X.-F. Ding S.-S. (2015). Erchen Decoction Prevents High-Fat Diet Induced Metabolic Disorders in C57BL/6 Mice. Evidence-Based Complement. Altern. Med. 2015, 1–9. 10.1155/2015/501272 PMC4609407 26504476

[B32] Gong S. Ye T. Wang M. Wang M. Li Y. Ma L. (2020). Traditional Chinese Medicine Formula Kang Shuai Lao Pian Improves Obesity, Gut Dysbiosis, and Fecal Metabolic Disorders in High-Fat Diet-Fed Mice. Front. Pharmacol. 11, 297. 10.3389/fphar.2020.00297 32269525 PMC7109517

[B33] Goodrich J. K. Waters J. L. Poole A. C. Sutter J. L. Koren O. Blekhman R. (2014). Human Genetics Shape the Gut Microbiome. Cell 159 (4), 789–799. 10.1016/j.cell.2014.09.053 25417156 PMC4255478

[B34] Habegger K. M. Al-Massadi O. Heppner K. M. Myronovych A. Holland J. Berger J. (2014). Duodenal Nutrient Exclusion Improves Metabolic Syndrome and Stimulates Villus Hyperplasia. Gut 63 (8), 1238–1246. 10.1136/gutjnl-2013-304583 24107591 PMC3981953

[B35] Hall A. B. Yassour M. Sauk J. Garner A. Jiang X. Arthur T. (2017). A Novel Ruminococcus Gnavus Clade Enriched in Inflammatory Bowel Disease Patients. Genome Med. 9 (1), 103. 10.1186/s13073-017-0490-5 29183332 PMC5704459

[B36] Hills R. Pontefract B. Mishcon H. Black C. Sutton S. Theberge C. (2019). Gut Microbiome: Profound Implications for Diet and Disease. Nutrients 11 (7), 1613. 10.3390/nu11071613 PMC6682904 31315227

[B37] Hsu C.-N. Hou C.-Y. Chang-Chien G.-P. Lin S. Tain Y.-L. (2020). Maternal N-Acetylcysteine Therapy Prevents Hypertension in Spontaneously Hypertensive Rat Offspring: Implications of Hydrogen Sulfide-Generating Pathway and Gut Microbiota. Antioxidants 9 (9), 856. 10.3390/antiox9090856 PMC7554905 32933169

[B38] Jang J.-H. Yeom M.-J. Ahn S. Oh J.-Y. Ji S. Kim T.-H. (2020). Acupuncture Inhibits Neuroinflammation and Gut Microbial Dysbiosis in a Mouse Model of Parkinson's Disease. Brain Behav. Immun. 89, 641–655. 10.1016/j.bbi.2020.08.015 32827699

[B39] Joffin N. Paschoal V. A. Gliniak C. M. Crewe C. Elnwasany A. Szweda L. I. (2021). Mitochondrial Metabolism Is a Key Regulator of the Fibro-Inflammatory and Adipogenic Stromal Subpopulations in white Adipose Tissue. Cell Stem Cell 28 (4), 702–717. 10.1016/j.stem.2021.01.002 33539722 PMC8026685

[B40] Kaakoush N. O. (2015). Insights into the Role of *Erysipelotrichaceae* in the Human Host. Front. Cel. Infect. Microbiol. 5, 84. 10.3389/fcimb.2015.00084 PMC4653637 26636046

[B41] Kassinen A. Krogius-Kurikka L. Mäkivuokko H. Rinttilä T. Paulin L. Corander J. (2007). The Fecal Microbiota of Irritable Bowel Syndrome Patients Differs Significantly from that of Healthy Subjects. Gastroenterology 133 (1), 24–33. 10.1053/j.gastro.2007.04.005 17631127

[B42] Keskitalo A. Munukka E. Toivonen R. Hollmén M. Kainulainen H. Huovinen P. (2018). *Enterobacter cloacae* Administration Induces Hepatic Damage and Subcutaneous Fat Accumulation in High-Fat Diet Fed Mice. PLoS One 13 (5), e0198262. 10.1371/journal.pone.0198262 29847581 PMC5976205

[B43] Kim J.-Y. Kwon Y. M. Kim I.-S. Kim J.-A. Yu D.-Y. Adhikari B. (2018). Effects of the Brown Seaweed Laminaria Japonica Supplementation on Serum Concentrations of IgG, Triglycerides, and Cholesterol, and Intestinal Microbiota Composition in Rats. Front. Nutr. 5, 23. 10.3389/fnut.2018.00023 29707542 PMC5906548

[B44] Kim K. N. Yao Y. Ju S. Y. (2019). Short Chain Fatty Acids and Fecal Microbiota Abundance in Humans with Obesity: A Systematic Review and Meta-Analysis. Nutrients 11 (10), 2512. 10.3390/nu11102512 PMC6835694 31635264

[B45] Kimura I. Ozawa K. Inoue D. Imamura T. Kimura K. Maeda T. (2013). The Gut Microbiota Suppresses Insulin-Mediated Fat Accumulation via the Short-Chain Fatty Acid Receptor GPR43. Nat. Commun. 4, 1829. 10.1038/ncomms2852 23652017 PMC3674247

[B46] Kindt A. Liebisch G. Clavel T. Haller D. Hörmannsperger G. Yoon H. (2018). The Gut Microbiota Promotes Hepatic Fatty Acid Desaturation and Elongation in Mice. Nat. Commun. 9 (1), 3760. 10.1038/s41467-018-05767-4 30218046 PMC6138742

[B47] Kovatcheva-Datchary P. Nilsson A. Akrami R. Lee Y. S. De Vadder F. Arora T. (2015). Dietary Fiber-Induced Improvement in Glucose Metabolism Is Associated with Increased Abundance of *Prevotella* . Cel Metab. 22 (6), 971–982. 10.1016/j.cmet.2015.10.001 26552345

[B48] Krautkramer K. A. Fan J. Bäckhed F. (2020). Gut Microbial Metabolites as Multi-Kingdom Intermediates. Nat. Rev. Microbiol. 19, 77–94. 10.1038/s41579-020-0438-4 32968241

[B49] Kusminski C. M. Bickel P. E. Scherer P. E. (2016). Targeting Adipose Tissue in the Treatment of Obesity-Associated Diabetes. Nat. Rev. Drug Discov. 15 (9), 639–660. 10.1038/nrd.2016.75 27256476

[B50] Larsen J. M. (2017). The Immune Response toPrevotellabacteria in Chronic Inflammatory Disease. Immunology 151 (4), 363–374. 10.1111/imm.12760 28542929 PMC5506432

[B51] Leclercq S. Matamoros S. Cani P. D. Neyrinck A. M. Jamar F. Stärkel P. (2014). Intestinal Permeability, Gut-Bacterial Dysbiosis, and Behavioral Markers of Alcohol-Dependence Severity. Proc. Natl. Acad. Sci. USA 111 (42), E4485–E4493. 10.1073/pnas.1415174111 25288760 PMC4210345

[B52] Lee A. Y. Park W. Kang T.-W. Cha M. H. Chun J. M. (2018). Network Pharmacology-Based Prediction of Active Compounds and Molecular Targets in Yijin-Tang Acting on Hyperlipidaemia and Atherosclerosis. J. Ethnopharmacology 221, 151–159. 10.1016/j.jep.2018.04.027 29698773

[B53] Lee S. M. Lee J. Kang E. Kim H.-L. Hwang G.-S. Jung J. (2020). Lipidomic Analysis Reveals Therapeutic Effects of Yijin-Tang on High-Fat/high-Cholesterol Diet-Induced Obese Mice. Phytomedicine 74, 152936. 10.1016/j.phymed.2019.152936 31088684

[B54] Li G. Xie C. Lu S. Nichols R. G. Tian Y. Li L. (2017). Intermittent Fasting Promotes White Adipose Browning and Decreases Obesity by Shaping the Gut Microbiota. Cel Metab. 26 (4), 672–685. 10.1016/j.cmet.2017.08.019 PMC5668683 28918936

[B55] Li J. Lin S. Vanhoutte P. M. Woo C. W. Xu A. (2016). Akkermansia Muciniphila Protects against Atherosclerosis by Preventing Metabolic Endotoxemia-Induced Inflammation in Apoe −/− Mice. Circulation 133 (24), 2434–2446. 10.1161/CIRCULATIONAHA.115.019645 27143680

[B56] Li L. Pan M. Pan S. Li W. Zhong Y. Hu J. (2020). Effects of Insoluble and Soluble Fibers Isolated from Barley on Blood Glucose, Serum Lipids, Liver Function and Caecal Short-Chain Fatty Acids in Type 2 Diabetic and normal Rats. Food Chem. Toxicol. 135, 110937. 10.1016/j.fct.2019.110937 31682932

[B57] Lippert K. Kedenko L. Antonielli L. Kedenko I. Gemeier C. Leitner M. (2017). Gut Microbiota Dysbiosis Associated with Glucose Metabolism Disorders and the Metabolic Syndrome in Older Adults. Beneficial Microbes 8 (4), 545–556. 10.3920/BM2016.0184 28701081

[B58] Liu C. Li J. Zhang Y. Philip A. Shi E. Chi X. (2015). Influence of Glucose Fermentation on CO2 Assimilation to Acetate in Homoacetogen Blautia Coccoides GA-1. J. Ind. Microbiol. Biotechnol. 42 (9), 1217–1224. 10.1007/s10295-015-1646-1 26153502

[B59] Luo J. Huang L. Wang A. Liu Y. Cai R. Li W. (2018). Resistin-Induced Endoplasmic Reticulum Stress Contributes to the Impairment of Insulin Signaling in Endothelium. Front. Pharmacol. 9, 1226. 10.3389/fphar.2018.01226 30416448 PMC6212567

[B60] Majchrzak M. Brzecka A. Daroszewski C. Błasiak P. Rzechonek A. Tarasov V. V. (2019). Increased Pain Sensitivity in Obese Patients after Lung Cancer Surgery. Front. Pharmacol. 10, 626. 10.3389/fphar.2019.00626 31258474 PMC6586739

[B61] Makki K. Deehan E. C. Walter J. Bäckhed F. (2018). The Impact of Dietary Fiber on Gut Microbiota in Host Health and Disease. Cell Host & Microbe 23 (6), 705–715. 10.1016/j.chom.2018.05.012 29902436

[B62] Maruvada P. Leone V. Kaplan L. M. Chang E. B. (2017). The Human Microbiome and Obesity: Moving beyond Associations. Cell Host & Microbe 22 (5), 589–599. 10.1016/j.chom.2017.10.005 29120742

[B63] Massier L. Chakaroun R. Tabei S. Crane A. Didt K. D. Fallmann J. (2020). Adipose Tissue Derived Bacteria Are Associated with Inflammation in Obesity and Type 2 Diabetes. Gut 69 (10), 1796–1806. 10.1136/gutjnl-2019-320118 32317332

[B64] Matthews D. R. Hosker J. P. Rudenski A. S. Naylor B. A. Treacher D. F. Turner R. C. (1985). Homeostasis Model Assessment: Insulin Resistance and ?-cell Function from Fasting Plasma Glucose and Insulin Concentrations in Man. Diabetologia 28 (7), 412–419. 10.1007/BF00280883 3899825

[B65] Müller M. Hernández M. A. G. Goossens G. H. Reijnders D. Holst J. J. Jocken J. W. E. (2019). Circulating but Not Faecal Short-Chain Fatty Acids Are Related to Insulin Sensitivity, Lipolysis and GLP-1 Concentrations in Humans. Sci. Rep. 9 (1), 12515. 10.1038/s41598-019-48775-0 31467327 PMC6715624

[B66] Muthuramalingam K. Singh V. Choi C. Choi S. I. Kim Y. M. Unno T. (2020). Dietary Intervention Using (1,3)/(1,6)-β-Glucan, a Fungus-Derived Soluble Prebiotic Ameliorates High-Fat Diet-Induced Metabolic Distress and Alters Beneficially the Gut Microbiota in Mice Model. Eur. J. Nutr. 59 (6), 2617–2629. 10.1007/s00394-019-02110-5 31664519

[B67] Ottosson F. Brunkwall L. Ericson U. Nilsson P. M. Almgren P. Fernandez C. (2018). Connection between BMI-Related Plasma Metabolite Profile and Gut Microbiota. J. Clin. Endocrinol. Metab. 103 (4), 1491–1501. 10.1210/jc.2017-02114 29409054

[B68] Ozato N. Saito S. Yamaguchi T. Katashima M. Tokuda I. Sawada K. (2019). Blautia Genus Associated with Visceral Fat Accumulation in Adults 20-76 Years of Age. NPJ Biofilms Microbiomes 5, 28. 10.1038/s41522-019-0101-x 31602309 PMC6778088

[B69] Park J. H. Seo I. Shim H. m. Cho H. (2020). Melatonin Ameliorates SGLT2 Inhibitor‐induced Diabetic Ketoacidosis by Inhibiting Lipolysis and Hepatic Ketogenesis in Type 2 Diabetic Mice. J. Pineal Res. 68 (2), e12623. 10.1111/jpi.12623 31743484

[B70] Pedersen H. K. Gudmundsdottir V. Nielsen H. B. Hyotylainen T. Nielsen T. Jensen B. A. H. (2016). Human Gut Microbes Impact Host Serum Metabolome and Insulin Sensitivity. Nature 535 (7612), 376–381. 10.1038/nature18646 27409811

[B71] Petersen L. M. Bautista E. J. Nguyen H. Hanson B. M. Chen L. Lek S. H. (2017). Community Characteristics of the Gut Microbiomes of Competitive Cyclists. Microbiome 5 (1), 98. 10.1186/s40168-017-0320-4 28797298 PMC5553673

[B72] Plovier H. Everard A. Druart C. Depommier C. Van Hul M. Geurts L. (2017). A Purified Membrane Protein from Akkermansia Muciniphila or the Pasteurized Bacterium Improves Metabolism in Obese and Diabetic Mice. Nat. Med. 23 (1), 107–113. 10.1038/nm.4236 27892954

[B73] Quan L.-H. Zhang C. Dong M. Jiang J. Xu H. Yan C. (2020). Myristoleic Acid Produced by Enterococci Reduces Obesity through Brown Adipose Tissue Activation. Gut 69 (7), 1239–1247. 10.1136/gutjnl-2019-319114 31744910

[B74] Rajilić-Stojanović M. Jonkers D. M. Salonen A. Hanevik K. Raes J. Jalanka J. (2015). Intestinal Microbiota and Diet in IBS: Causes, Consequences, or Epiphenomena?. Am. J. Gastroenterol. 110 (2), 278–287. 10.1038/ajg.2014.427 25623659 PMC4317767

[B75] Ramette A. (2007). Multivariate Analyses in Microbial Ecology. FEMS Microbiol. Ecol. 62 (2), 142–160. 10.1111/j.1574-6941.2007.00375.x 17892477 PMC2121141

[B76] Reichardt N. Duncan S. H. Young P. Belenguer A. McWilliam Leitch C. Scott K. P. (2014). Phylogenetic Distribution of Three Pathways for Propionate Production within the Human Gut Microbiota. ISME J. 8 (6), 1323–1335. 10.1038/ismej.2014.14 24553467 PMC4030238

[B77] Roden M. Shulman G. I. (2019). The Integrative Biology of Type 2 Diabetes. Nature 576 (7785), 51–60. 10.1038/s41586-019-1797-8 31802013

[B78] Rodriguez J. Hiel S. Neyrinck A. M. Le Roy T. Pötgens S. A. Leyrolle Q. (2020). Discovery of the Gut Microbial Signature Driving the Efficacy of Prebiotic Intervention in Obese Patients. Gut 69 (11), 1975–1987. 10.1136/gutjnl-2019-319726 32041744 PMC7569399

[B79] Rothschild D. Weissbrod O. Barkan E. Kurilshikov A. Korem T. Zeevi D. (2018). Environment Dominates over Host Genetics in Shaping Human Gut Microbiota. Nature 555 (7695), 210–215. 10.1038/nature25973 29489753

[B80] Rumberger J. M. Arch J. R. S. Green A. (2014). Butyrate and Other Short-Chain Fatty Acids Increase the Rate of Lipolysis in 3T3-L1 Adipocytes. PeerJ 2, e611. 10.7717/peerj.611 25320679 PMC4193401

[B81] Saltiel A. R. Olefsky J. M. (2017). Inflammatory Mechanisms Linking Obesity and Metabolic Disease. J. Clin. Invest. 127 (1), 1–4. 10.1172/JCI92035 28045402 PMC5199709

[B82] Sanna S. van Zuydam N. R. Mahajan A. Kurilshikov A. Vich Vila A. Võsa U. (2019). Causal Relationships Among the Gut Microbiome, Short-Chain Fatty Acids and Metabolic Diseases. Nat. Genet. 51 (4), 600–605. 10.1038/s41588-019-0350-x 30778224 PMC6441384

[B83] Scherer T. O'Hare J. Diggs-Andrews K. Schweiger M. Cheng B. Lindtner C. (2011). Brain Insulin Controls Adipose Tissue Lipolysis and Lipogenesis. Cel Metab. 13 (2), 183–194. 10.1016/j.cmet.2011.01.008 PMC3061443 21284985

[B84] Schwiertz A. Taras D. Schäfer K. Beijer S. Bos N. A. Donus C. (2010). Microbiota and SCFA in Lean and Overweight Healthy Subjects. Obesity (Silver Spring) 18 (1), 190–195. 10.1038/oby.2009.167 19498350

[B85] Schwimmer J. B. Johnson J. S. Angeles J. E. Behling C. Belt P. H. Borecki I. (2019). Microbiome Signatures Associated with Steatohepatitis and Moderate to Severe Fibrosis in Children with Nonalcoholic Fatty Liver Disease. Gastroenterology 157 (4), 1109–1122. 10.1053/j.gastro.2019.06.028 31255652 PMC6756995

[B86] Segata N. Izard J. Waldron L. Gevers D. Miropolsky L. Garrett W. S. (2011). Metagenomic Biomarker Discovery and Explanation. Genome Biol. 12 (6), R60. 10.1186/gb-2011-12-6-r60 21702898 PMC3218848

[B87] Seo S.-H. Unno T. Park S.-E. Kim E.-J. Lee Y.-M. Na C.-S. (2019). Korean Traditional Medicine (Jakyakgamcho-Tang) Ameliorates Colitis by Regulating Gut Microbiota. Metabolites 9 (10), 226. 10.3390/metabo9100226 PMC6835967 31615012

[B88] Shi Y. Kellingray L. Zhai Q. Gall G. L. Narbad A. Zhao J. (2018). Structural and Functional Alterations in the Microbial Community and Immunological Consequences in a Mouse Model of Antibiotic-Induced Dysbiosis. Front. Microbiol. 9, 1948. 10.3389/fmicb.2018.01948 30186263 PMC6110884

[B89] Shiau M.-Y. Chuang P.-H. Yang C.-P. Hsiao C.-W. Chang S.-W. Chang K.-Y. (2019). Mechanism of Interleukin-4 Reducing Lipid Deposit by Regulating Hormone-Sensitive Lipase. Sci. Rep. 9 (1), 11974. 10.1038/s41598-019-47908-9 31427606 PMC6700157

[B90] Si J. You H. J. Yu J. Sung J. Ko G. (2017). *Prevotella* as a Hub for Vaginal Microbiota under the Influence of Host Genetics and Their Association with Obesity. Cell Host & Microbe 21 (1), 97–105. 10.1016/j.chom.2016.11.010 28017660

[B91] Stanislawski M. A. Dabelea D. Wagner B. D. Sontag M. K. Lozupone C. A. Eggesbø M. (2017). Pre-pregnancy Weight, Gestational Weight Gain, and the Gut Microbiota of Mothers and Their Infants. Microbiome 5 (1), 113. 10.1186/s40168-017-0332-0 28870230 PMC5584478

[B92] Terzo S. Mulè F. Caldara G. F. Baldassano S. Puleio R. Vitale M. (2020). Pistachio Consumption Alleviates Inflammation and Improves Gut Microbiota Composition in Mice Fed a High-Fat Diet. Ijms 21 (1), 365. 10.3390/ijms21010365 PMC6981517 31935892

[B93] Tirosh A. Calay E. S. Tuncman G. Claiborn K. C. Inouye K. E. Eguchi K. (2019). The Short-Chain Fatty Acid Propionate Increases Glucagon and FABP4 Production, Impairing Insulin Action in Mice and Humans. Sci. Transl. Med. 11 (489), eaav0120. 10.1126/scitranslmed.aav0120 31019023

[B94] Tremaroli V. Bäckhed F. (2012). Functional Interactions between the Gut Microbiota and Host Metabolism. Nature 489 (7415), 242–249. 10.1038/nature11552 22972297

[B95] Truong D. T. Tett A. Pasolli E. Huttenhower C. Segata N. (2017). Microbial Strain-Level Population Structure and Genetic Diversity from Metagenomes. Genome Res. 27 (4), 626–638. 10.1101/gr.216242.116 28167665 PMC5378180

[B96] Tuovinen E. Keto J. Nikkilä J. Mättö J. Lähteenmäki K. (2013). Cytokine Response of Human Mononuclear Cells Induced by Intestinal Clostridium Species. Anaerobe 19, 70–76. 10.1016/j.anaerobe.2012.11.002 23168133

[B97] Turnbaugh P. J. Ley R. E. Mahowald M. A. Magrini V. Mardis E. R. Gordon J. I. (2006). An Obesity-Associated Gut Microbiome with Increased Capacity for Energy Harvest. Nature 444 (7122), 1027–1031. 10.1038/nature05414 17183312

[B98] Udayappan S. Manneras-Holm L. Chaplin-Scott A. Belzer C. Herrema H. Dallinga-Thie G. M. (2016). Oral Treatment with Eubacterium Hallii Improves Insulin Sensitivity in Db/db Mice. NPJ Biofilms Microbiomes 2, 16009. 10.1038/npjbiofilms.2016.9 28721246 PMC5515273

[B99] van den Munckhof I. C. L. Kurilshikov A. Ter Horst R. Riksen N. P. Joosten L. A. B. Zhernakova A. (2018). Role of Gut Microbiota in Chronic Low-Grade Inflammation as Potential Driver for Atherosclerotic Cardiovascular Disease: a Systematic Review of Human Studies. Obes. Rev. 19 (12), 1719–1734. 10.1111/obr.12750 30144260

[B100] Virtue A. T. McCright S. J. Wright J. M. Jimenez M. T. Mowel W. K. Kotzin J. J. (2019). The Gut Microbiota Regulates white Adipose Tissue Inflammation and Obesity via a Family of microRNAs. Sci. Transl. Med. 11 (496), eaav1892. 10.1126/scitranslmed.aav1892 31189717 PMC7050429

[B101] Wang J. Wang P. Li D. Hu X. Chen F. (2020). Beneficial Effects of Ginger on Prevention of Obesity through Modulation of Gut Microbiota in Mice. Eur. J. Nutr. 59 (2), 699–718. 10.1007/s00394-019-01938-1 30859364

[B102] Wang Y. Fei Y. Liu L. Xiao Y. Pang Y. Kang J. (2018). Polygonatum Odoratum Polysaccharides Modulate Gut Microbiota and Mitigate Experimentally Induced Obesity in Rats. Ijms 19 (11), 3587. 10.3390/ijms19113587 PMC6274832 30428630

[B103] Wei X. Tao J. Xiao S. Jiang S. Shang E. Zhu Z. (2018). Xiexin Tang Improves the Symptom of Type 2 Diabetic Rats by Modulation of the Gut Microbiota. Sci. Rep. 8 (1), 3685. 10.1038/s41598-018-22094-2 29487347 PMC5829262

[B104] Yin C. Liu W. h. Liu Y. Wang L. Xiao Y. (2019). PID1 Alters the Antilipolytic Action of Insulin and Increases Lipolysis via Inhibition of AKT/PKA Pathway Activation. PLoS One 14 (4), e0214606. 10.1371/journal.pone.0214606 30990811 PMC6467375

[B105] Ze X. Duncan S. H. Louis P. Flint H. J. (2012). Ruminococcus Bromii Is a keystone Species for the Degradation of Resistant Starch in the Human colon. ISME J. 6 (8), 1535–1543. 10.1038/ismej.2012.4 22343308 PMC3400402

[B106] Zhang H. Ta N. Chen P. Wang H. (2017). Erchen Decoction and Linguizhugan Decoction Ameliorate Hepatic Insulin Resistance by Inhibiting IRS-1Ser307 Phosphorylation *In Vivo* and *In Vitro* . Evidence-Based Complement. Altern. Med. 2017, 1–11. 10.1155/2017/1589871 PMC5467344 28630632

[B107] Zhang L. Zhou W. Zhan L. Hou S. Zhao C. Bi T. (2020a). Fecal Microbiota Transplantation Alters the Susceptibility of Obese Rats to Type 2 Diabetes Mellitus. Aging 12 (17), 17480–17502. 10.18632/aging.103756 32920548 PMC7521520

[B108] Zhang M. Shao Y. Gao B. Chen J. Zhang P. Hu Y. (2020b). Erchen Decoction Mitigates Lipid Metabolism Disorder by the Regulation of PPARγ and LPL Gene in a High-Fat Diet C57BL/6 Mice Model. Evidence-Based Complement. Altern. Med. 2020, 1–8. 10.1155/2020/9102475 PMC7085882 32256662

[B109] Zhang M. Zhu J. Zhang X. Zhao D.-g. Ma Y.-y. Li D. (2020c). Aged Citrus Peel (Chenpi) Extract Causes Dynamic Alteration of Colonic Microbiota in High-Fat Diet Induced Obese Mice. Food Funct. 11 (3), 2667–2678. 10.1039/c9fo02907a 32159537

[B110] Zhang X. Zhao S. Song X. Jia J. Zhang Z. Zhou H. (2018). Inhibition Effect of glycyrrhiza Polysaccharide (GCP) on Tumor Growth through Regulation of the Gut Microbiota Composition. J. Pharmacol. Sci. 137 (4), 324–332. 10.1016/j.jphs.2018.03.006 30150145

[B111] Zhou D. Pan Q. Xin F.-Z. Zhang R.-N. He C.-X. Chen G.-Y. (2017). Sodium Butyrate Attenuates High-Fat Diet-Induced Steatohepatitis in Mice by Improving Gut Microbiota and Gastrointestinal Barrier. Wjg 23 (1), 60–75. 10.3748/wjg.v23.i1.60 28104981 PMC5221287

[B112] Zhou W. Xu H. Zhan L. Lu X. Zhang L. (2019). Dynamic Development of Fecal Microbiome during the Progression of Diabetes Mellitus in Zucker Diabetic Fatty Rats. Front. Microbiol. 10, 232. 10.3389/fmicb.2019.00232 30837966 PMC6382700

[B113] Zhu L. Baker S. S. Gill C. Liu W. Alkhouri R. Baker R. D. (2013). Characterization of Gut Microbiomes in Nonalcoholic Steatohepatitis (NASH) Patients: a Connection between Endogenous Alcohol and NASH. Hepatology 57 (2), 601–609. 10.1002/hep.26093 23055155

[B114] Zimmermann R. Strauss J. G. Haemmerle G. Schoiswohl G. Birner-Gruenberger R. Riederer M. (2004). Fat Mobilization in Adipose Tissue Is Promoted by Adipose Triglyceride Lipase. Science 306 (5700), 1383–1386. 10.1126/science.1100747 15550674

