# Supplementary material for: The Effects of Erchen Decoction on Gut Microbiota and Lipid Metabolism Disorders in Zucker Diabetic Fatty Rats
Source: Front Pharmacol. 2021 Jul 22;12:647529. doi: 10.3389/fphar.2021.647529 (PMC8339961; doi:10.3389/fphar.2021.647529)
Supplement: Supplementary file 1 [file DataSheet1.docx]

***Supplementary Material***

The effects of Erchen Decoction on gut microbiota and lipid metabolism disorders in Zucker diabetic fatty rats

**Tian Zhao^1^, Libin Zhan^1*^, Wen Zhou^1^, Wanxin Chen^1^, Jintong Luo^1^, Lijing Zhang^1^, Zebin Weng^1^, Chunyan Zhao^1^, Shenlin Liu^2, 3*^**

*** Correspondence:**

Libin Zhan: [zlbnj@njucm.edu.cn](mailto:zlbnj@njucm.edu.cn)

Shenlin Liu: [lsljsszyy@126.com](mailto:lsljsszyy@126.com)

## Supplementary Figures

**
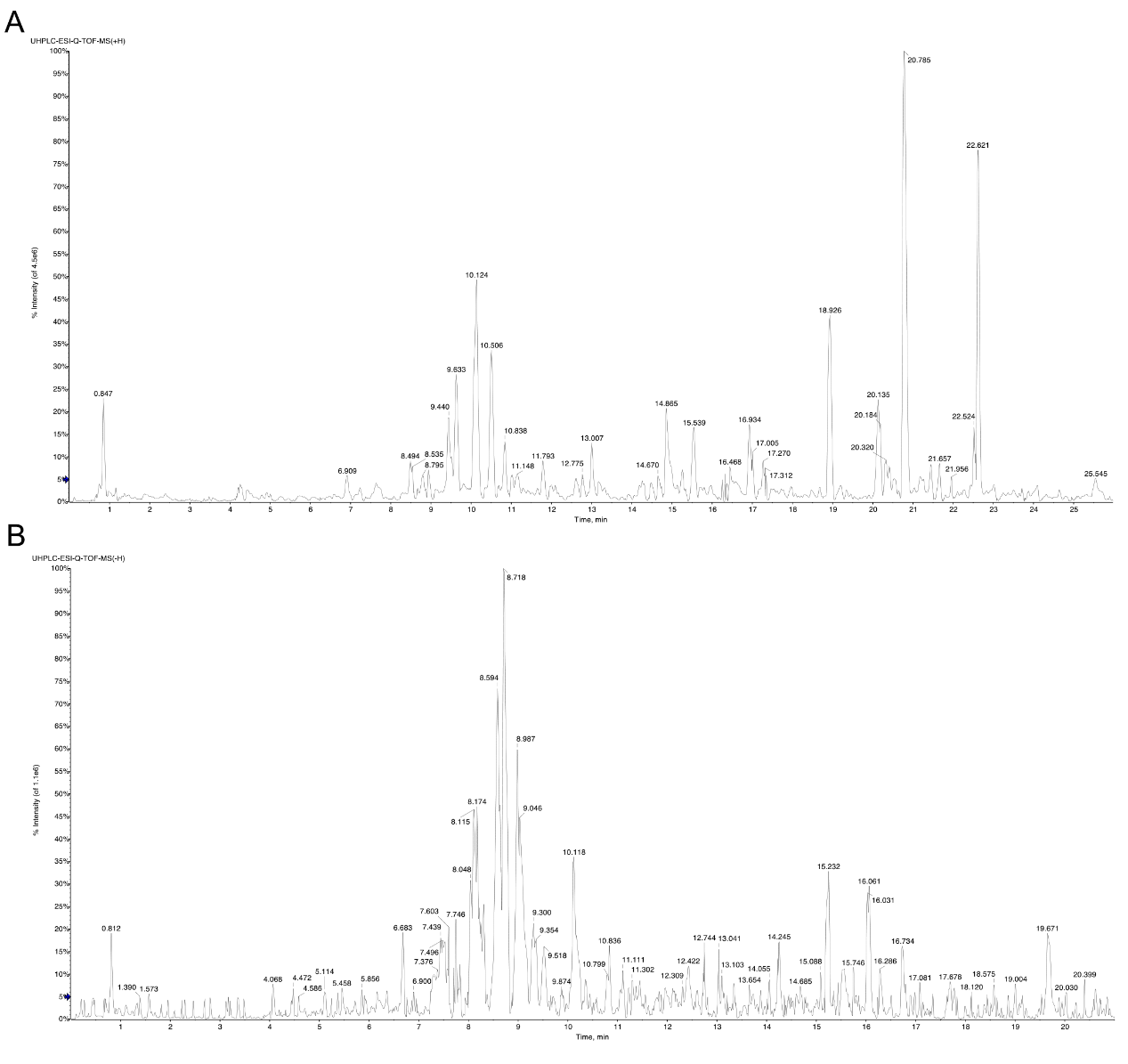
Supplementary Figure 1.** UHPLC-ESI-Q-TOF-MS total ion chromatogram of ECD sample. **(A)** Positive ion mode. **(B)** Negative ion mode.


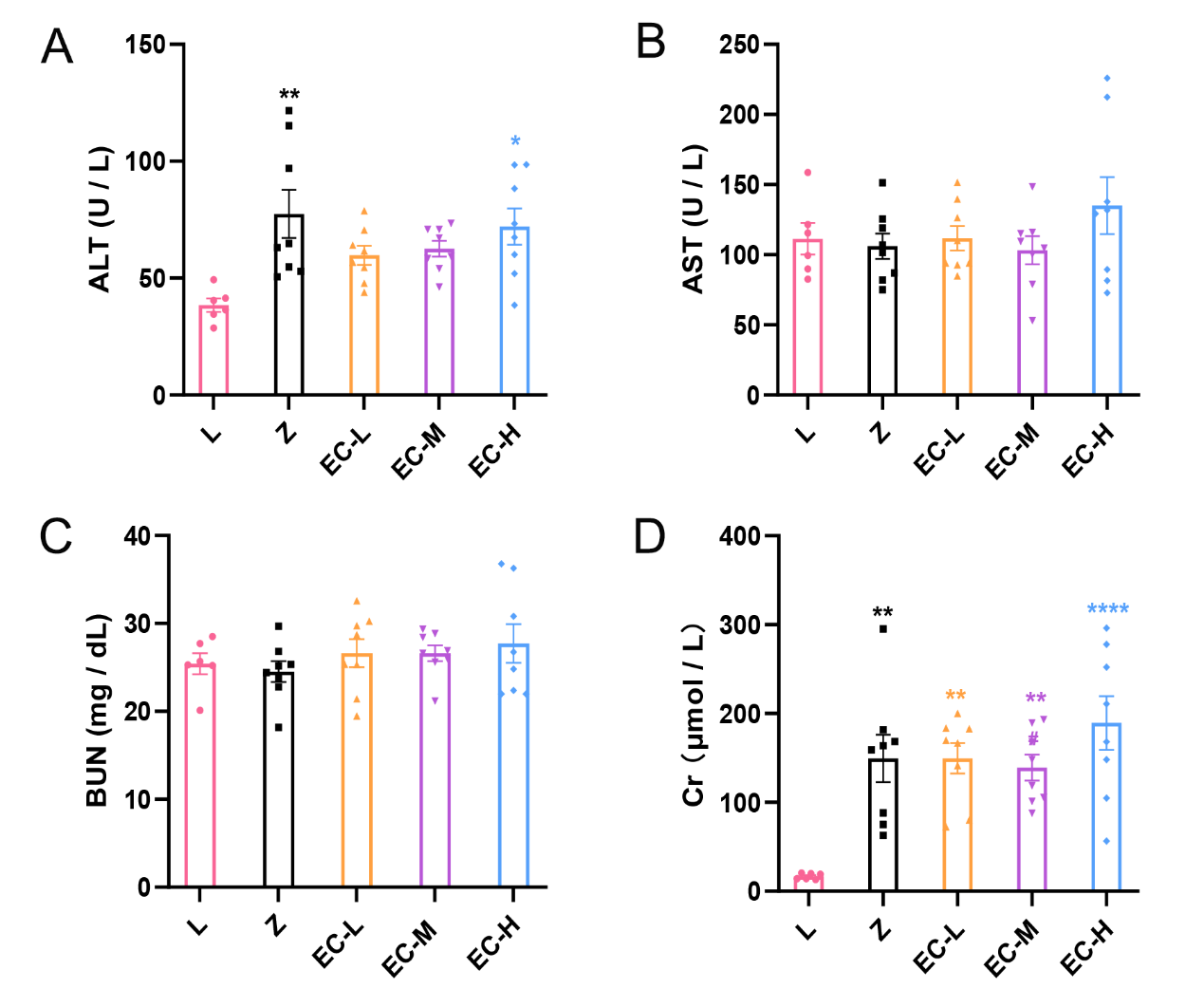


**Supplementary Figure 2.** The effect of ECD on the liver and kidney function of ZDF rats. (A) ALT. (B) AST. (C) BUN. (D) Cr. Data are expressed as means ± SEM (n=6-8. Z, EC-L, EC-M, and EC-H vs. L, ^*^*P* < 0.05, ^**^*P* < 0.01, ^****^*P* < 0.0001).


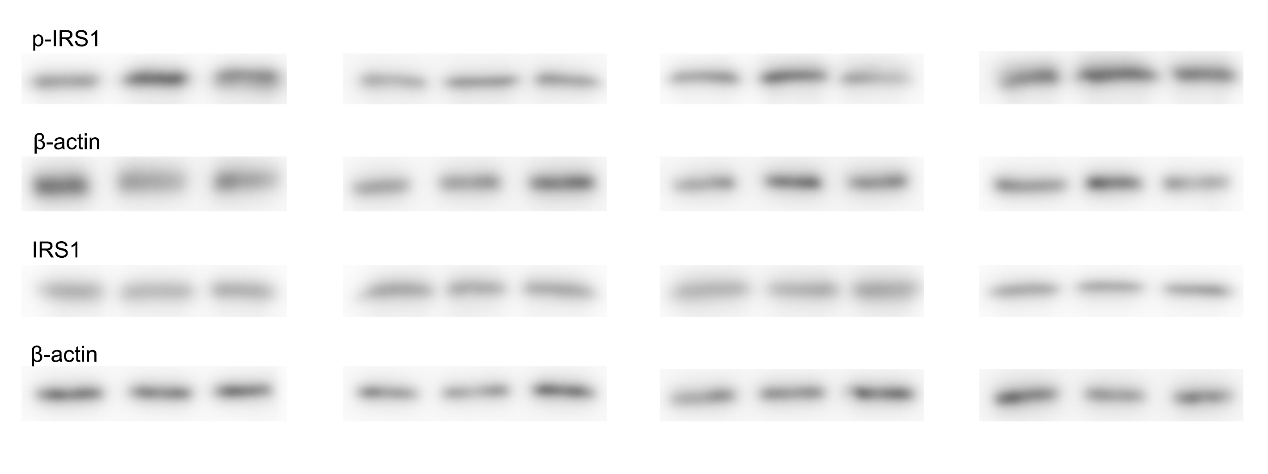


**Supplementary Figure 3.** Original bands of western blotting for p-IRS1/IRS1 in Fig. 3C.


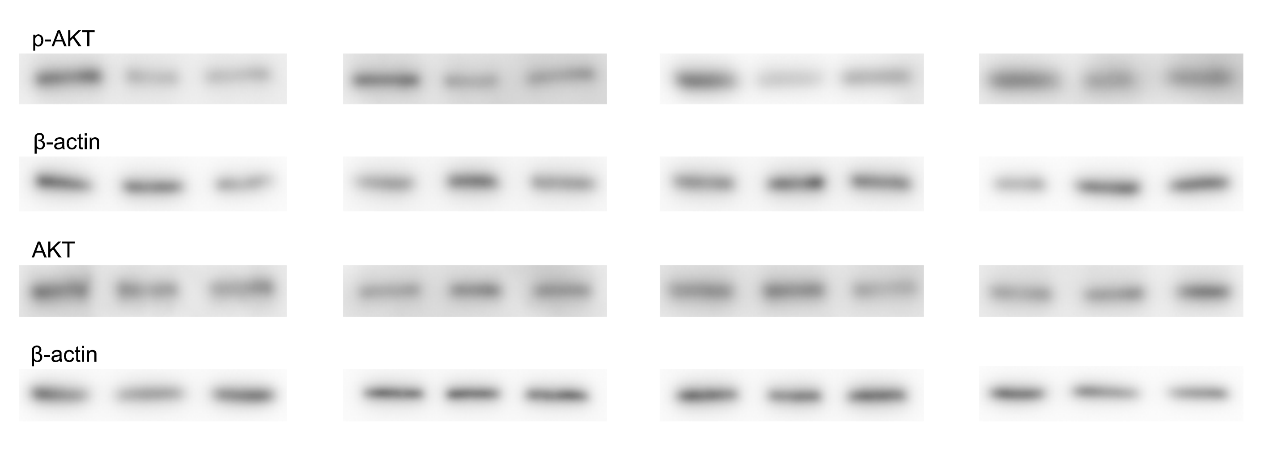
**Supplementary Figure 4.** Original bands of western blotting for p-AKT/AKT in Fig. 3C.


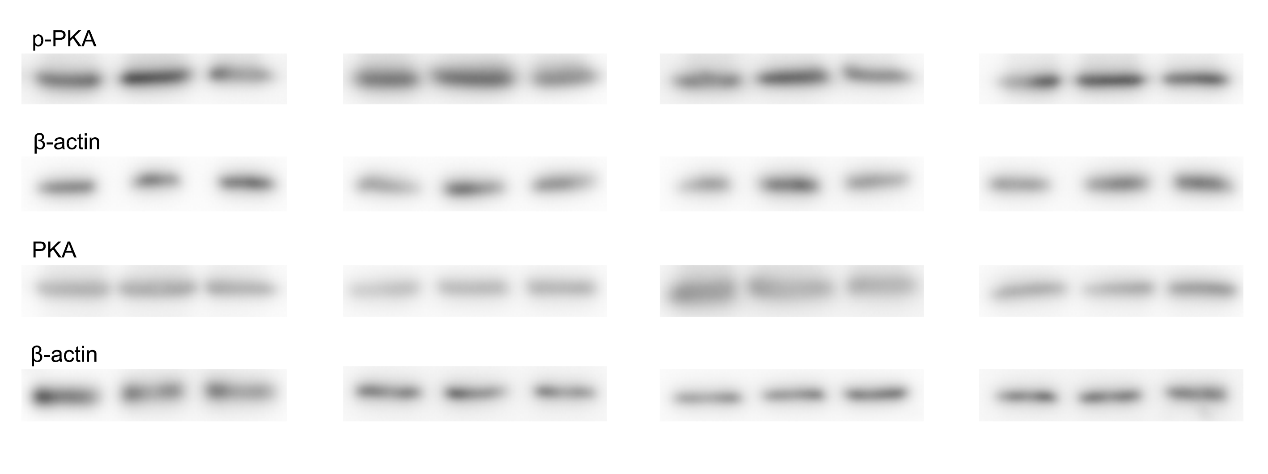


**Supplementary Figure 5.** Original bands of western blotting for p-PKA/PKA in Fig. 3C.


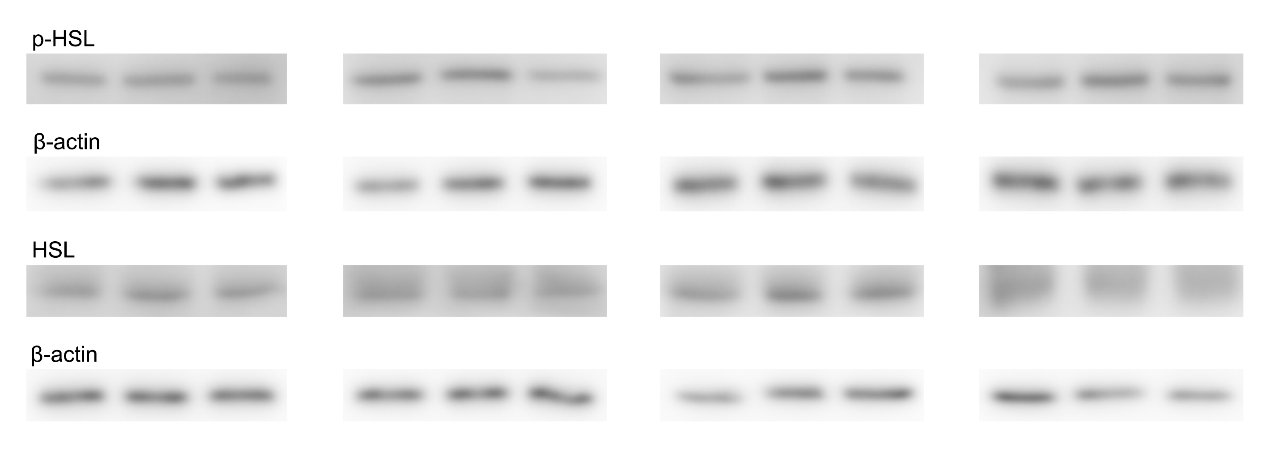


**Supplementary Figure 6.** Original bands of western blotting for p-HSL/HSL in Fig. 3C.


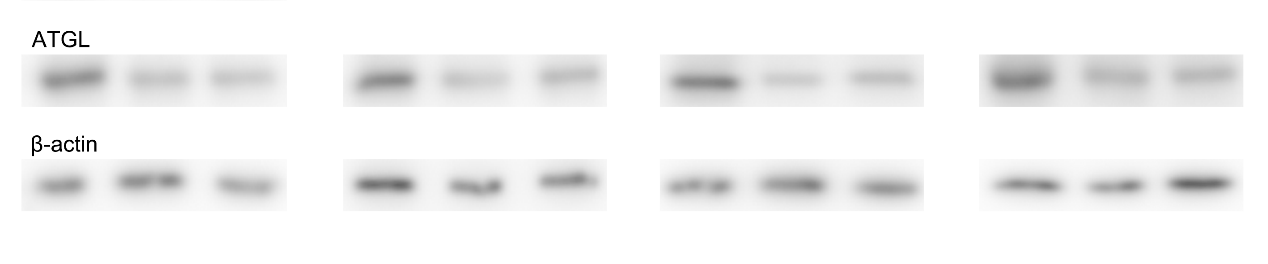
**Supplementary Figure 7**. Original bands of western blotting for ATGL in Fig. 3C.

## Supplementary Tables

## Supplementary Table 1. Diet composition table of ZL and ZDF rats.

|  | MD17121, Medicience | Purina #5008, Lab diet |
| --- | --- | --- |
| Crude protein, % | ≥20.0 | ≥23.0 |
| Crude fat, % | ≥6.0 | ≥6.5 |
| Crude fiber, % | ≤5.0 | ≤4.0 |
| Ash, % | ≤8.0 | ≤8.0 |
| Calcium, % | 1.0-1.8 | 1.20 |
| Phosphorus, % | 0.6-1.2 | 0.65 |
| Moisture content, % | ≤10.0 |  |
| Lysine, % | ≥1.32 |  |
| Methionine + Cystine, % | ≥0.78 |  |
| Glutamic Acid, % |  | 4.98 |
| Arginine, % |  | 1.53 |

**Supplementary Table 2.** The chemical composition detected of ECD sample by UHPLC-ESI-Q-TOF-MS (in positive and negative ion mode). The detection was performed on the Shimadzu LC10ATVP high performance liquid chromatograph (Shimadzu Corporation, Japan) combined with the AB Sciex TripleTOF 5600+ Time-of-Flight Mass Spectrometer (AB SCIEX, Foster City, CA, USA). The separation was performed on an ACQUITY UPLC BEH C18 column (100 mm × 2.1 mm, 1.7 μm), the column temperature was 40°C and injection volume was 2 μL. The mobile phase was 0.1% formic acid aqueous solution (A)-acetonitrile (B), gradient elution: 0→15min, 5%B→50%B; 15→20 min, 50%B→80%B; 20→25min, 80%B→80%B; the flow rate was 0.4 mL / min. The MS conditions were set to: positive and negative ion mode; CUR: 35.000psi; GS1: 55.000psi; GS2: 55.000psi; TEM: 550.000°C; CE: -10.000V (negative ion mode); DP: -60.000V (negative ion mode); scan Range m/z 100-1000 and 50-1000.

| Serial number | Retention time (min) | Mass spectrum information | | | | Chemical composition name |
| --- | --- | --- | --- | --- | --- | --- |
|  |  | Mass [M+H] | Calc. Mass | Error (Da) | Fragment peak |  |
| 1 | 12.78 | 985.46424 | 985.4639 | 0.4 | 615.3869, 453.3322 | Licoricesaponin A3 |
| 2 | 14.19 | 839.40826 | 839.406 | 2.7 | 487.3432, 469.3329 | Licoricesaponin G2 |
| 3 | 14.85 | 823.41304 | 823.4111 | 2.4 | 453.3356 | Glycyrrhizic acid |
| 4 | 14.19 | 821.3962 | 821.3954 | 1 | 451.32 | Licoricesaponin E2 |
| 5 | 7.24 | 595.16632 | 595.1658 | 1 | 577.155, 409.0947 | Vicenin 2 |
| 6 | 9.64 | 581.18722 | 581.1865 | 1.3 | 273.0758 | Naringin |
| 7 | 8.1 | 565.15554 | 565.1552 | 0.6 | 547.1413, 529.1166, 529.1383, 499.1148 | Isoschaftoside |
| 8 | 8.1 | 565.15554 | 565.1552 | 0.6 | 547.1413, 529.1166, 529.1383, 499.1148 | Schaftoside |
| 9 | 10.72 | 563.17596 | 563.1759 | 0.1 | 269.0799 | Glycyroside |
| 10 | 8.92 | 551.17628 | 551.1759 | 0.7 | 257.0818 | Licuraside |
| 11 | 8.92 | 551.17628 | 551.1759 | 0.7 | 257.0818 | Isoliquiritin apioside |
| 12 | 8.92 | 551.17628 | 551.1759 | 0.7 | 257.0818 | Liquiritin apioside |
| 13 | 10.82 | 515.22739 | 515.2276 | -0.3 | 469.2213, 455.2021, 413.1872 | Nomilin |
| 14 | 14.86 | 471.34725 | 471.3469 | 0.8 | none | Glycyrrhetinic acid |
| 15 | 15.65 | 471.20171 | 471.2013 | 0.8 | 425.1944 | Limonin |
| 16 | 17.98 | 437.19391 | 437.1959 | -4.5 | none | Kanzonol K |
| 17 | 16.97 | 433.14977 | 433.1493 | 1.1 | 403.1026 | 3, 3', 4', 5, 6, 7, 8-Heptamethoxyflavone |
| 18 | 11.02 | 431.1334 | 431.1337 | -0.6 | 269.0798 | Isoononin |
| 19 | 19.58 | 425.23261 | 425.2323 | 0.8 | 369.1849, 369.1622, 369.1323, 313.1235, 313.1056, 313.0896, 313.0682, 221.115, 191.1077, 165.0563 | Kanzonol H |
| 20 | 19.58 | 425.23261 | 425.2323 | 0.8 | 369.1849, 369.1622, 369.1323, 313.1235, 313.1056, 313.0896, 313.0682, 221.115, 191.1077, 165.0563 | Licoricidin |
| 21 | 9.64 | 419.13382 | 419.1337 | 0.4 | 401.1966, 401.1244, 383.1101, 315.0987, 315.091, 315.0764,  263.055, 257.0789, 165.0185, 147.0629, 147.0415, 129.0536, 85.0268 | Isoliquiritin |
| 22 | 9.64 | 419.13382 | 419.1337 | 0.4 | 401.1966, 401.1244, 383.1101, 315.0987, 315.091, 273.0746, 257.0789, 165.0185, 147.0629, 147.0415, 129.0536, 71.0485,  69.0334 | Neoisoliquiritigenin |
| 23 | 9.64 | 419.13382 | 419.1337 | 0.4 | 401.1966, 401.1244, 383.1101, 315.0987, 315.091, 315.0764, 257.0789, 165.0185, 147.0629, 147.0415, 71.0485, 69.0334 | Liquiritin |
| 24 | 9.64 | 419.13382 | 419.1337 | 0.4 | 401.1966, 401.1244, 383.1101, 315.0987, 315.091, 315.0764, 273.0746, 257.0789, 165.0185, 147.0629, 147.0415, 71.0485,  69.0334 | Neoliquiritin |
| 25 | 16.31 | 403.13918 | 403.1387 | 1.1 | 373.0911 | Nobiletin |
| 26 | 18.78 | 385.1647 | 385.1646 | 0.3 | 367.0649, 329.0957, 339.1231, 339.1112, 311.1161, 247.0931 | Kanzonol N |
| 27 | 18.78 | 385.1647 | 385.1646 | 0.3 | 367.0649, 329.0957, 339.1231, 339.1112, 311.1161, 279.0074, 267.0374, 267.0101, 266.9862, 247.0931 | Glicoisoflavanone |
| 28 | 17.77 | 383.1492 | 383.1489 | 0.7 | 327.0864 | Kanzonol O |
| 29 | 17.77 | 383.1492 | 383.1489 | 0.7 | 327.0864 | Licoricone |
| 30 | 17.77 | 383.1492 | 383.1489 | 0.7 | 327.0864 | Glycyrin |
| 31 | 12.9 | 375.18054 | 375.1802 | 0.9 | 375.1766, 375.0934, 357.1582, 356.8731, 177.0931, 163.0718, 137.0592 | Hexahydrocurcumin |
| 32 | 17.32 | 373.1285 | 373.1282 | 0.9 | 343.0808 | Sinensetin |
| 33 | 17.32 | 373.1285 | 373.1282 | 0.9 | 343.0808 | Tangeretin |
| 34 | 17.32 | 373.1285 | 373.1282 | 0.9 | 343.0808 | 5, 7, 3', 4', 5'-Pentamethoxyflavone |
| 35 | 17.32 | 373.1285 | 373.1282 | 0.9 | 343.0808 | Isosinensetin |
| 36 | 19.33 | 371.18559 | 371.1853 | 0.8 | 315.1355, 315.1194, 303.1216, 235.1333, 167.0706, 149.0593, 123.0424, 69.069 | Glyasperin D |
| 37 | 19.33 | 371.18559 | 371.1853 | 0.8 | 315.1355, 315.1194, 303.1216, 235.1333, 181.0905, 167.0706, 149.0593, 123.0424, 69.069 | Kanzonol R |
| 38 | 16.83 | 369.13359 | 369.1333 | 0.9 | 313.0722, 285.0778 | Glycycoumarin |
| 39 | 16.83 | 369.13359 | 369.1333 | 0.9 | 313.0722, 285.0778 | Isoglycycoumarin |
| 40 | 16.83 | 369.13359 | 369.1333 | 0.9 | 313.0722, 285.0778 | Glycyrrhisoflavanone |
| 41 | 16.83 | 369.13359 | 369.1333 | 0.9 | 313.0722, 285.0778 | Gancaonin B |
| 42 | 16.83 | 369.13359 | 369.1333 | 0.9 | 313.0722, 285.0778 | Gancaonin N |
| 43 | 16.83 | 369.13359 | 369.1333 | 0.9 | 313.0722, 285.0778 | Glisoflavone |
| 44 | 16.83 | 369.13359 | 369.1333 | 0.9 | 313.0722, 285.0778 | 5, 2', 4'-Trihydroxy-7-methoxy-6-prenylisoflavone |
| 45 | 16.83 | 369.13359 | 369.1333 | 0.9 | none | Curcumin |
| 46 | 18.03 | 367.11791 | 367.1176 | 0.8 | 311.0556 | Isoglycyrol |
| 47 | 18.03 | 367.11791 | 367.1176 | 0.8 | 311.0556 | Glycyrol |
| 48 | 18.75 | 359.14912 | 359.1489 | 0.6 | none | Glicophenone |
| 49 | 13.75 | 359.11275 | 359.1125 | 0.6 | none | 4'-Hydroxy-5, 6, 7, 8-tetramethoxyflavone |
| 50 | 13.75 | 359.11275 | 359.1125 | 0.6 | none | 6-Hydroxyluteolin 5, 6, 3', 4'-tetramethyl ether |
| 51 | 13.75 | 359.11275 | 359.1125 | 0.6 | none | 5-Hydroxy-6, 7, 3', 4'-tetramethoxyflavone |
| 52 | 17.05 | 357.16996 | 357.1697 | 0.9 | 338.9212, 301.1107, 301.0773, 221.1198, 191.1084, 165.0537, 153.056, 135.0488, 135.0417, 123.0421 | Glyasperin C |
| 53 | 17.05 | 357.16996 | 357.1697 | 0.9 | 338.9212, 301.1107, 301.0773, 221.1198, 191.1084, 165.0537, 153.056, 135.0488, 135.0417, 123.0421 | Gingerenone A |
| 54 | 17.15 | 355.15411 | 355.154 | 0.3 | 299.0912, 299.0566 | Arizonicanol E |
| 55 | 17.15 | 355.15411 | 355.154 | 0.3 | 299.0912, 299.0566 | Gancaonin I |
| 56 | 17.15 | 355.15411 | 355.154 | 0.3 | 299.0912, 299.0566 | Licobenzofuran |
| 57 | 17.15 | 355.15411 | 355.154 | 0.3 | 299.0912, 299.0566 | 3'-Methoxyglabridin |
| 58 | 17.64 | 355.11795 | 355.1176 | 1 | 299.0559 | Gancaonin L |
| 59 | 17.64 | 355.11795 | 355.1176 | 1 | 299.0559 | Isolicoflavonol |
| 60 | 17.64 | 355.11795 | 355.1176 | 1 | 299.0559 | Gancaonin O |
| 61 | 17.64 | 355.11795 | 355.1176 | 1 | none | Gancaonin C |
| 62 | 17.64 | 355.11795 | 355.1176 | 1 | 299.0559 | Glycyrrhisoflavone |
| 63 | 17.64 | 355.11795 | 355.1176 | 1 | 299.0559 | Licoflavonol |
| 64 | 17.64 | 355.11795 | 355.1176 | 1 | 299.0559 | Licoisoflavanone |
| 65 | 23.88 | 353.26794 | 353.2686 | -2 | 291.2327, 99.0694, 99.0615, 73.0466, 59.0309 | [10]-Gingerdiol |
| 66 | 18.67 | 353.13828 | 353.1384 | -0.2 | 38.9629 | Gancaonin G |
| 67 | 18.67 | 353.13828 | 353.1384 | -0.2 | 189.0925, 153.0515, 38.9629 | Gancaonin A |
| 68 | 18.67 | 353.13828 | 353.1384 | -0.2 | 189.0925, 153.0515, 38.9629 | Gancanin M |
| 69 | 16.82 | 351.12295 | 351.1227 | 0.7 | 295.0611, 265.0528 | Glycyrrhizol B |
| 70 | 15.31 | 343.11803 | 343.1176 | 1.2 | 313.0704 | 5, 7, 3', 4'-Tetramethoxyflavone |
| 71 | 11.23 | 341.1381 | 341.1384 | -0.7 | 322.8974, 311.1327 | 8-Prenylnaringenin |
| 72 | 11.23 | 341.1381 | 341.1384 | -0.7 | 322.8974, 311.1327 | Licocoumarone |
| 73 | 17.78 | 339.15941 | 339.1591 | 0.9 | none | Gancaonin X |
| 74 | 17.78 | 339.15941 | 339.1591 | 0.9 | none | 4'-O-Methylglabridin |
| 75 | 20.44 | 333.24258 | 333.2424 | 0.5 | 177.0901, 137.0599 | 10-Shogaol |
| 76 | 12.81 | 329.10279 | 329.102 | 2.5 | 310.9261,299.0531,248.9338,  174.9896 | Salvigenin |
| 77 | 12.81 | 329.10279 | 329.102 | 2.5 | 310.9261,299.0531,248.9338,  174.9896 | Isoscutellarein 7, 8, 4'-trimethyl ether |
| 78 | 18.36 | 325.14363 | 325.1434 | 0.6 | 189.0892, 149.0589, 136.9303, 123.0427 | Isobavachalcone |
| 79 | 20.64 | 323.22201 | 323.2217 | 1 | 277.1837, 221.1296, 221.1137, 193.1195, 175.1189, 175.1075, 133.0976, 129.0629, 129.0534, 101.0226, 85.027, 57.0693,  57.0336, | (S)-8-Gingerol |
| 80 | 21.81 | 307.22699 | 307.2268 | 0.7 | 260.9651, 195.1043, 149.0602, 115.0682, 107.05, 57.0697 | [8]-Paradol |
| 81 | 18.74 | 305.21128 | 305.2111 | 0.5 | 177.0908, 175.0754, 137.0596 | [8]-Shogaol |
| 82 | 10.16 | 303.08609 | 303.0863 | -0.7 | 287.1003, 177.0547, 153.0178 | 3, 4, 3', 4'-Tetrahydroxy-2-methoxychalcone |
| 83 | 10.16 | 303.08609 | 303.0863 | -0.7 | 287.1003, 177.0547, 153.0178 | Carpusin |
| 84 | 16.44 | 277.18028 | 277.1798 | 1.6 | 177.0914, 137.0594 | [6]-Shogaol |
| 85 | 16.36 | 275.16413 | 275.1642 | -0.2 | 256.9234, 229.141, 229.1212, 201.1303, 191.0667, 187.1112, 173.0962, 159.1128, 149.0965, 136.9331, 133.1006, 131.0861, 109.0266 | [6]-Dehydroshogaol |
| 86 | 9.64 | 273.07598 | 273.0758 | 0.9 | 153.0162, 147.0442 | Naringenin |
| 87 | 13.17 | 271.09664 | 271.0965 | 0.6 | 229.0848, 177.0577, 123.0424, 121.0261, 107.0485 | Echinatin |
| 88 | 14.66 | 269.08095 | 269.0808 | 0.4 | none | Formononetin |
| 89 | 8.96 | 257.08126 | 257.0808 | 1.6 | 147.0455, 137.0247 | Liquiritigenin |
| 90 | 8.96 | 257.08126 | 257.0808 | 1.6 | 147.0455, 137.0247 | Isoliquiritigenin |
| 91 | 18.24 | 219.17462 | 219.1743 | 1.3 | 203.1437 | beta-Sinensal |
| 92 | 18.24 | 219.17462 | 219.1743 | 1.3 | 203.1437 | Zerumbone |
| 93 | 18.24 | 219.17462 | 219.1743 | 1.3 | 203.1437 | alpha-Sinensal |
| 94 | 15.29 | 205.19522 | 205.1951 | 0.7 | 149.134, 149.1235, 149.0232, 147.0199, 135.1253, 135.1137, 69.0687, 57.0707, 55.051,  39.0232 | Zonarene |
| 95 | 15.29 | 205.19522 | 205.1951 | 0.7 | 149.134, 149.1235, 149.0232, 135.1253, 135.1137, 123.1238, 123.1128, 122.9511, 109.0983, 107.0834, 105.0705, 104.9401, 95.0842, 93.0698, 81.0706,  79.0543, 69.0687, 57.0707, 55.051,39.0232 | beta-Bisabolene |
| 96 | 15.29 | 205.19522 | 205.1951 | 0.7 | 149.134, 149.1235, 149.0232, 140.9581, 135.1253, 135.1137, 123.1238, 123.1128, 122.9511, 109.0983, 107.0834, 105.0705, 104.9401, 95.0842, 93.0698,  81.0706, 79.0543, 69.0687,  57.0707, 55.051, 39.0232 | (-)-Zingiberene |
| 97 | 15.29 | 205.19522 | 205.1951 | 0.7 | 149.134, 149.1235, 149.0232, 135.1253, 135.1137, 123.1238, 123.1128, 122.9511, 109.0983, 107.0834, 105.0705, 104.9401, 95.0842, 93.0698, 81.0706, 69.0687, 57.0707, 55.051, 39.0232 | (E, E)-alpha-Farnesene |
| 98 | 15.29 | 205.19522 | 205.1951 | 0.7 | 149.134, 149.1235, 149.0232, 140.9581, 135.1253, 135.1137, 123.1238, 123.1128, 122.9511, 109.0983, 107.0834, 105.0705, 104.9401, 95.0842, 93.0698,  81.0706, 79.0543, 69.0687,  57.0707, 55.051, 39.0232 | beta-Sesquiphellandrene |
| 99 | 15.29 | 205.19522 | 205.1951 | 0.7 | 149.134, 149.1235, 149.0232, 147.0199, 135.1253, 135.1137, 123.1238, 123.1128, 122.9511, 109.0983, 95.0842, 93.0698,  69.0687, 57.0707, 55.051, 39.0232 | gamma-Cadinene |
| 100 | 15.29 | 205.19522 | 205.1951 | 0.7 | 149.134, 149.1235, 149.0232, 135.1253, 135.1137, 123.1238, 123.1128, 122.9511, 109.0983, 107.0834, 105.0705, 104.9401, 95.0842, 93.0698, 81.0706,  79.0543, 69.0687, 57.0707,  55.051, 39.0232 | Sesquithujene |
| 101 | 15.29 | 205.19522 | 205.1951 | 0.7 | 149.134, 149.1235, 149.0232，147.0199, 135.1253, 135.1137, 109.0983, 95.0842, 93.0698,  69.0687, 57.0707, 55.051,  39.0232 | alpha-Muurolene |
| 102 | 15.29 | 205.19522 | 205.1951 | 0.7 | 205.1883, 205.1556, 149.134, 149.1235, 149.0232, 147.0199, 135.1253, 135.1137, 123.1238, 123.1128, 122.9511, 69.0687,  57.0707, 55.051 | (+)-Cyclosativene |
| 103 | 15.29 | 205.19522 | 205.1951 | 0.7 | 149.134, 149.1235, 149.0232, 147.0199, 135.1253, 135.1137, 123.1238, 123.1128, 122.9511, 109.0983, 107.0834, 95.0842,  81.0706, 69.0687, 57.0707, 55.051 | gamma-Elemene |
| 104 | 15.29 | 205.19522 | 205.1951 | 0.7 | 149.134, 149.1235, 149.0232, 147.0199, 135.1253, 135.1137, 123.1238, 123.1128, 122.9511, 109.0983, 107.0834, 95.0842,  81.0706, 69.0687, 57.0707, 55.051 | delta-Elemene |
| 105 | 15.29 | 205.19522 | 205.1951 | 0.7 | 149.134, 149.1235, 149.0232, 135.1253, 135.1137, 123.1238, 123.1128, 122.9511, 109.0983, 107.0834, 105.0705, 104.9401, 95.0842, 93.0698, 81.0706, 69.0687, 57.0707, 55.051, 39.0232 | (E)-beta-Farnesene |
| 106 | 15.29 | 205.19522 | 205.1951 | 0.7 | 149.134, 149.1235, 149.0232, 147.0199, 135.1253, 135.1137, 123.1238, 123.1128, 122.9511, 109.0983, 107.0834, 95.0842,  81.0706, 69.0687, 57.0707, 55.051 | beta-Elemene |
| 107 | 15.29 | 205.19522 | 205.1951 | 0.7 | 149.134, 149.1235, 149.0232, 135.1253, 135.1137, 123.1238, 123.1128, 122.9511, 109.0983, 107.0834, 105.0705, 104.9401, 95.0842, 93.0698, 81.0706,  79.0543, 69.0687, 57.0707, 55.051, 39.0232 | beta-Bisabolene |
| 108 | 15.29 | 205.19522 | 205.1951 | 0.7 | 149.134, 149.1235, 149.0232, 135.1253, 135.1137, 123.1238, 123.1128, 122.9511, 109.0983, 107.0834, 95.0842, 81.0706,  69.0687, 57.0707, 55.051, 39.0232 | Germacrene B |
| 109 | 15.29 | 205.19522 | 205.1951 | 0.7 | 149.134, 149.1235, 149.0232, 147.0199, 135.1253, 135.1137, 123.1238, 123.1128, 122.9511, 109.0983, 107.0834, 95.0842,  81.0706, 69.0687, 57.0707, 55.051, 39.0232 | alpha-Selinene |
| 110 | 15.29 | 205.19522 | 205.1951 | 0.7 | 149.134, 149.1235, 149.0232, 147.0199, 135.1253, 135.1137, 123.1238, 123.1128, 122.9511, 109.0983, 95.0842, 93.0698,  69.0687, 57.0707, 55.051, 39.0232 | gamma-Muurolene |
| 111 | 15.29 | 205.19522 | 205.1951 | 0.7 | 149.134, 149.1235, 149.0232, 147.0199, 135.1253, 135.1137, 123.1238, 123.1128, 122.9511, 109.0983, 107.0834, 95.0842,  81.0706, 69.0687, 57.0707, 55.051, 39.0232 | beta-Selinene |
| 112 | 15.29 | 205.19522 | 205.1951 | 0.7 | 149.134, 149.1235, 149.0232, 147.0199, 135.1253, 135.1137, 123.1238, 123.1128, 122.9511, 69.0687, 57.0707, 55.051, 39.0232 | Copaene |
| 113 | 15.29 | 205.19522 | 205.1951 | 0.7 | 149.134, 149.1235, 149.0232, 147.0199, 135.1253, 135.1137, 109.0983, 95.0842, 93.0698,  69.0687, 57.0707, 55.051, 39.0232 | beta-Cadinene |
| 114 | 15.29 | 205.19522 | 205.1951 | 0.7 | 149.134, 149.1235, 149.0232, 147.0199, 57.0707, 55.051, 39.0232 | alpha-Cedrene |
| 115 | 15.29 | 205.19522 | 205.1951 | 0.7 | 149.134, 149.1235, 149.0232, 147.0199, 135.1253, 135.1137, 123.1238, 123.1128, 122.9511, 109.0983, 107.0834, 95.0842,  93.0698, 81.0706, 69.0687, 57.0707, 55.051, 39.0232 | beta-Caryophyllene |
| 116 | 8.79 | 193.04945 | 193.0495 | -0.4 | 175.0166, 147.019, 137.0645, 118.9904, 72.9844, 57.0335, 44.9914 | Scopoletin |
| 117 | 5.4 | 163.07518 | 163.0754 | -1.1 | 135.0806, 123.0834, 116.9895, 116.9757, 45.0322 | Safrole |
| 118 | 8.44 | 153.1271 | 153.1274 | -1.9 | 107.0847, 106.9907, 81.0674,  70.9775, 43.017 | (E)-Citral |
| 119 | 8.44 | 153.1271 | 153.1274 | -1.9 | 44.9905, 43.017 | Camphor |
| 120 | 8.44 | 153.1271 | 153.1274 | -1.9 | 107.0847, 106.9907, 81.0674,  70.9775, 43.017 | (Z)-Citral |
| 121 | 8.44 | 153.1271 | 153.1274 | -1.9 | 107.0847, 106.9907, 81.0674,  70.9775, 43.017 | (E)-Citral |
| 122 | 8.44 | 153.1271 | 153.1274 | -1.9 | 107.0847, 106.9907, 95.0838,  93.0685, 81.0674, 72.9851,  70.9775, 44.9905, 43.017 | p-Mentha-1, 8-dien-6-ol |
| 123 | 8.44 | 153.1271 | 153.1274 | -1.9 | 107.0847, 106.9907,  95.0838,  93.0685, 81.0674, 70.9775,  43.017 | cis-Ocimene |
| 124 | 8.44 | 153.1271 | 153.1274 | -1.9 | 107.0847, 106.9907, 81.0674,  70.9775, 43.017 | Citral |
| 125 | 8.45 | 135.11667 | 135.1168 | -1.2 | 91.0543, 79.0535, 78.9979,  77.0383, 44.9916 | p-Cymene |
| 126 | 8.45 | 135.11667 | 135.1168 | -1.2 | 91.0543, 79.0535, 78.9979,  77.0383 | 2, 6-Dimethyl-1, 3, 5, 7-octatetraene |

| Serial number | Retention time (min) | Mass spectrum information | | | | Chemical composition name |
| --- | --- | --- | --- | --- | --- | --- |
|  |  | Mass  [M-H] | Calc. Mass | Error (Da) | Fragment peak |  |
| 1 | 13.31 | 485.32614 | 485.32615 | 0 | none | Poricoic acid B |
| 2 | 14.66 | 483.34648 | 483.34689 | -0.8 | none | Polyporenic acid C |
| 3 | 9.16 | 463.1235 | 463.12349 | 0 | none | Mumenin |
| 4 | 9.9 | 433.14923 | 433.14931 | -0.2 | none | 3,3',4',5,6,7,8-Heptamethoxyflavone |
| 5 | 13.57 | 381.13368 | 381.13327 | 1.1 | 351.0868 | 4'-Hydroxy-3,6-dimethoxy-6'',6''-dimethylpyrano[2,3:7,8]flavone |
| 6 | 12.69 | 369.13352 | 369.13327 | 0.7 | 337.1058, 267.0301, 229.0841, 161.0232, 109.0285 | Curcumin |
| 7 | 0.79 | 361.09094 | 361.09179 | -2.4 | none | Acerosin |
| 8 | 0.79 | 361.09094 | 361.09179 | -2.4 | none | Sudachitin |
| 9 | 6.48 | 355.10315 | 355.10236 | 2.2 | 308.8903, 295.0809, 265.0745, 265.0654, 235.0599,218.945, 207.0654, 193.0503, 191.0696, 175.0376, 149.0755 | Chlorogenic acid |
| 10 | 16.6 | 347.22 | 347.22169 | -4.9 | none | 1-Dehydro- [10]-gingerdione |
| 11 | 17.18 | 323.22067 | 323.22169 | -3.2 | none | (S)-8-Gingerol |
| 12 | 13.45 | 309.20714 | 309.20604 | 3.6 | 209.1544, 99.0078 | [7]-Gingerol |
| 13 | 16.37 | 307.22795 | 307.22677 | 3.8 | none | [8]-Paradol |
| 14 | 15.77 | 305.2111 | 305.21112 | -0.1 | none | [8]-Shogaol |
| 15 | 8.76 | 301.07017 | 301.07066 | -1.6 | 151.0028, | Takakin |
| 16 | 14.22 | 293.21136 | 293.21112 | 0.8 | 275.2002, 249.1887, 235.1691, 221.1559, 205.1314, 205.122, 193.1589, 191.0672 | [7]-Paradol |
| 17 | 14.22 | 293.21136 | 293.21112 | 0.8 | 275.2002, 249.1887, 235.1691, 221.1559, 205.1314, 205.122, 193.1589, 191.0672 | Methyl [6]-paradol |
| 18 | 13.05 | 293.17577 | 293.17474 | 3.5 | 221.155 | [6]-Gingerdione |
| 19 | 14.6 | 277.18049 | 277.17982 | 2.4 | 233.19 | [6]-Shogaol |
| 20 | 13.52 | 195.13854 | 195.13796 | 3 | 109.0663 | Carvyl acetate |

**Supplementary Table 3.** The LDA score and *P*-value of genus that changed significantly during the development of obesity and formed a significant difference from group L at 9 weeks of age.

|  | Phylum | Genus | LDA Score | *P*-value | |  |
| --- | --- | --- | --- | --- | --- | --- |
| Gut microbiota increased gradually with the development of obesity | *Bacteroidetes* | *Prevotella* | 4.614 | | 1.629E-03 | |
|  | *Firmicutes* | *Blautia* | 4.420 | | 7.500E-04 | |
|  | *Firmicutes* | *Dorea* | 3.553 | | 1.216E-03 | |
|  | *Firmicutes* | *SMB53* | 3.508 | | 3.301E-02 | |
|  | *Firmicutes* | *Allobaculum* | 3.175 | | 5.590E-04 | |
|  | *Firmicutes* | *Coprobacillus* | 3.155 | | 6.770E-04 | |
|  | *Firmicutes* | [*Ruminococcus*] | 3.129 | | 3.515E-02 | |
|  | *Firmicutes* | *Holdemania* | 3.016 | | 2.533E-03 | |
|  | *Proteobacteria* | *Sutterella* | 2.926 | | 2.077E-02 | |
| Gut microbiota reduced gradually with the development of obesity | *Verrucomicrobia* | *Akkermansia* | 3.641 | | 1.066E-02 | |
|  | *Firmicutes* | *Oscillospira* | 3.462 | | 2.086E-02 | |
|  | *Actinobacteria* | *Adlercreutzia* | 3.350 | | 1.578E-03 | |
|  | *Firmicutes* | *Dehalobacterium* | 3.139 | | 2.043E-02 | |
|  | *Firmicutes* | *f_Erysipelotrichaceae-g_Clostridium* | 3.091 | | 1.564E-02 | |

**Supplementary Table 4.** The LDA score and *P*-value of genus with significant difference among 3 groups due to ECD intervention at 9 weeks of age in Fig. 5E.

| Phylum | Genus | L vs. Z | | Z vs. EC | |
| --- | --- | --- | --- | --- | --- |
|  |  | LDA score | *P*-value | LDA score | *P*-value |
| *Bacteroidetes* | *Prevotella* | 4.650 | 1.946E-03 | 4.542 | 6.323E-03 |
| *Firmicutes* | *Blautia* | 4.461 | 1.923E-03 | 4.344 | 4.574E-03 |
| *Firmicutes* | *Ruminococcus* | 3.919 | 4.509E-03 | 3.942 | 1.629E-03 |
| *Firmicutes* | *Holdemania* | 3.400 | 2.391E-03 | 2.896 | 5.053E-03 |
| *Firmicutes* | *Coprobacillus* | 3.288 | 6.118E-03 | 2.933 | 1.768E-02 |
| *Verrucomicrobia* | *Akkermansia* | 3.442 | 3.111E-02 | 3.223 | 3.776E-03 |
